# Subject‐specific muscle properties from diffusion tensor imaging significantly improve the accuracy of musculoskeletal models

**DOI:** 10.1111/joa.13261

**Published:** 2020-06-29

**Authors:** James P. Charles, Barbara Grant, Kristiaan D’Août, Karl T. Bates

**Affiliations:** ^1^ Department of Musculoskeletal and Ageing Science Institute of Life Course and Medical Sciences University of Liverpool Liverpool UK

**Keywords:** biomechanical modelling, fibre lengths, magnetic resonance imaging, muscle torques, muscle volumes

## Abstract

Musculoskeletal modelling is an important platform on which to study the biomechanics of morphological structures in vertebrates and is widely used in clinical, zoological and palaeontological fields. The popularity of this approach stems from the potential to non‐invasively quantify biologically important but difficult‐to‐measure functional parameters. However, while it is known that model predictions are highly sensitive to input values, it is standard practice to build models by combining musculoskeletal data from different sources resulting in ‘generic’ models for a given species. At present, there are little quantitative data on how merging disparate anatomical data in models impacts the accuracy of these functional predictions. This issue is addressed herein by quantifying the accuracy of both subject‐specific human limb models containing individualised muscle force‐generating properties and models built using generic properties from both elderly and young individuals, relative to experimental muscle torques obtained from an isokinetic dynamometer. The results show that subject‐specific models predict isokinetic muscle torques to a greater degree of accuracy than generic models at the ankle (root‐mean‐squared error – 7.9% vs. 49.3% in elderly anatomy‐based models), knee (13.2% vs. 57.3%) and hip (21.9% vs. 32.8%). These results have important implications for the choice of musculoskeletal properties in future modelling studies, and the relatively high level of accuracy achieved in the subject‐specific models suggests that such models can potentially address questions about inter‐subject variations of muscle functions. However, despite relatively high levels of overall accuracy, models built using averaged generic muscle architecture data from young, healthy individuals may lack the resolution and accuracy required to study such differences between individuals, at least in certain circumstances. The results do not wholly discourage the continued use of averaged generic data in musculoskeletal modelling studies but do emphasise the need for to maximise the accuracy of input values if studying intra‐species form–function relationships in the musculoskeletal system.

## Graphical abstract

Diffusion tensor imaging (DTI) allows for the accurate visualisation and measurement of important muscle architecture factors such as muscle fibre lengths in vivo, but the accuracy of these data derived from this method has not been tested in a functional context, such as in subject‐specific musculoskeletal models. This study aimed to test the relative accuracy of personalised muscle architecture data from DTI and MRI compared to more generic data at predicting muscle torques in subject‐specific musculoskeletal models. It was found that, on average, fully subject‐specific data predict muscle torques to a significantly greater degree of accuracy relative to generic data, although the extent of this is dependent on an individual subject's anthropometry, suggesting that in some cases, more generic muscle architecture data may be suitable for predicting complex muscle functions in musculoskeletal models if the methods for obtaining individualised data are not available.

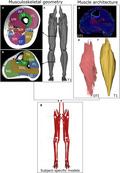

## 1 INTRODUCTION

Musculoskeletal modelling allows for detailed simulations and predictions of biomechanical performance during complex movements and behaviours (locomotion, feeding, etc.), which may be difficult or even impossible to replicate in experimental conditions. For this reason, it has become a widely used research tool across clinical (Falisse *et al*., 2019; Trinler *et al*., 2019), veterinary (Watson *et al*., 2014; Lerner *et al*., 2015; Becker *et al*., 2019), zoological (Sellers *et al*., 2013; Rankin *et al*., 2016; Goh *et al*., 2017; Charles *et al*., 2018; Ellis *et al*., 2018; Sellers and Hirasaki, 2018) and palaeontological fields (Sellers *et al*., 2005; Bates *et al*., 2012; Bates and Schachner, 2012; Crompton *et al*., 2012; Sellers *et al*., 2017; Bishop *et al*., 2018). However, with this popularity and desire to build increasingly complex models, there comes a necessity to fully optimise and validate such models. Across these fields, it is becoming recognised that, where possible, model predictions should be validated against experimental measures (Sellers *et al*., 2005; Sellers and Manning, 2007; Bates *et al*., 2012; Shi *et al*., 2012; Groning *et al*., 2013). This is necessary not only to validate the computational methods themselves (and their mathematical representations of mechanics and animal physiology), but also because musculoskeletal models are rarely constructed from homogeneous data sets where all aspects of morphology and physiology are measured from a single individual or even individuals with similar demographics or morphometry. Indeed, in zoological research, values for certain input parameters are often unavailable in the literature for the species under study, and as a result, values qualitatively considered ‘average’ for vertebrates in general are used (Bates *et al*., 2010; Hutchinson, 2012). In both circumstances, researchers have often conducted sensitivity analyses in acknowledgement of the potential for abstraction or inaccuracy in their model predictions (Sellers *et al*., 2005; Sellers and Manning, 2007; Bates *et al*., 2010; Bates *et al*., 2012; Groning *et al*., 2013). These sensitivity analyses have particularly highlighted the potential errors in performance predictions (muscle forces, running speeds, bite forces, etc.) related to parameters that underpin the force‐generating capacity of muscles, often referred to as muscle architecture (Hutchinson *et al*., 2007; Bates *et al*., 2010; Bates *et al*., 2012; Groning *et al*., 2013; Bates and Falkingham, 2018).

Clearly, the gold standard approach would be to construct a model or models in which all anatomical input data, including muscle architecture and musculoskeletal geometry (bones and muscle attachments), are measured in the individual or individuals being modelled, thus creating a highly subject‐specific modelling framework. However, such subject‐specific models are currently much more expensive and time‐consuming to produce relative to generic‐based or ‘averaged’ models and as such have rarely been used to this extent in any species. However, research into the validity of subject‐specific models of the human musculoskeletal system is becoming more widespread, as it is thought that such models may be more accurate for certain tasks than the often used generic or scaled generic models (Lenaerts *et al*., 2008; Scheys *et al*., 2008; Scheys *et al*., 2009; Scheys *et al*., 2011; Valente *et al*., 2014; Navacchia *et al*., 2016; Prinold *et al*., 2016; Dejtiar *et al*., 2020; Gu and Pandy, 2020; Modenese and Kohout, 2020; Nardini *et al*., 2020). For example, models with subject‐specific musculoskeletal geometry have been shown to be more effective for predicting muscle moment arms and joint contact forces, with respective differences of 36% (Scheys *et al*., 2008) and 0.61 xBW (Lenaerts *et al*., 2008) relative to generic models. Large differences in model outputs such as these could substantially affect the conclusions drawn from subject‐specific models and simulations relative to scaled generic equivalents and reaffirm the need for accurate and possibly individualised input data in musculoskeletal modelling studies.

While the inclusion of individualised musculoskeletal geometry has been readily shown to improve musculoskeletal models, the effect of including detailed subject‐specific muscle architecture data in particular is less clear. Within musculoskeletal models, the force‐generating capacity of individual musculotendon unit (MTU) actuators is usually represented with the standard Hill‐type model (Zajac, 1989), where force generation is calculated using four main input parameters: maximum isometric force (*F*_max_), optimal fibre length (*L*_f_), fibre pennation angles (θ) and tendon slack length (*L*_ts_). In traditional generic musculoskeletal models, these force‐generating properties are often obtained from a combination of published muscle architecture data sets (Arnold *et al*., 2010; Rajagopal *et al*., 2016). Many of these data sets contain important parameters (such as muscle fibre lengths) obtained from dissection studies, which usually include elderly cadaveric specimens due to difficulties in extensively obtaining similar data in vivo (Wickiewicz *et al*., 1983; Ward *et al*., 2009). However, because of known changes in muscle architecture and functional capability due to ageing (Narici *et al*., 1985; Narici *et al*., 2008; Moore *et al*., 2014), these properties are unlikely to be representative of the wider human population. While other muscle architecture parameters such as muscle masses and volumes (from which maximum force can be derived) have been commonly estimated from more representative populations using magnetic resonance imaging (MRI) or similar methods (Infantolino *et al*., 2007; Handsfield *et al*., 2014; Nijholt *et al*., 2017; Charles *et al*., 2019), estimating a muscle's potential maximum force output has traditionally relied on fibre properties such lengths and pennation angles from cadaveric studies. Concerns could therefore be raised over the applicability of many aspects of generic muscle architecture data for musculoskeletal modelling, particularly when studying stronger individuals or investigating subtle or complex between‐subject variations in muscle functions. This could also be particularly important in clinical contexts, for example if predictions of orthopaedic surgery outcomes or personalised rehabilitation protocols are being informed by outputs from musculoskeletal models and simulations (Arnold *et al*., 2006; Seth *et al*., 2018).

Several methods have been proposed for overcoming the assumed functional limitations of generic muscle architecture. Musculoskeletal model outputs have been shown to be highly sensitive to variations in muscle fibre lengths and tendon slack lengths in particular (Scovil and Ronsky, 2006; Redl *et al*., 2007; Ackland *et al*., 2012; Charles *et al*., 2016), with studies attempting to optimise these parameters based on a subject's anthropometry to reduce these errors (Winby *et al*., 2008; Modenese *et al*., 2016). Alternatively, muscle maximum isometric force values have been scaled to different individuals based on various metrics such as relative body mass (van der Krogt *et al*., 2016), a combination of relative body mass and relative musculotendon lengths (Correa and Pandy, 2011), regression equations (Handsfield *et al*., 2014) or dynamometer‐based approaches (Kainz *et al*., 2018). However, the lack of subject specificity of these scaled muscle force‐generating properties could present crucial errors if used in studies investigating detailed muscle dynamics.

Diffusion tensor imaging (DTI), a form of MRI, of skeletal muscle has recently emerged as a viable and repeatable method of obtaining muscle architecture *in vivo* (Bolsterlee *et al*., 2019; Charles *et al*., 2019), thereby circumventing traditional difficulties of obtaining estimates of individualised estimates of muscle force‐generating properties. The DTI technique has been shown to accurately visualise detailed in vivo anatomy within a variety of muscle groups (Froeling *et al*., 2012; Sieben *et al*., 2016; Bolsterlee *et al*., 2018) within individuals of a variety of ages and with various pathologies (Malis *et al*., 2019; Sahrmann *et al*., 2019), as well as to build a novel data set of in vivo muscle architecture from young, healthy individuals (Charles *et al*., 2019). However, despite its widespread use, how accurately these muscle data simulate individual muscle functions and overall biomechanical performance within subject‐specific musculoskeletal models relative to scaled or optimised generic data has not been tested.

Herein, these important issues surrounding model design and resolution are addressed by quantifying the absolute accuracy of both subject‐specific human limb models, containing individualised muscle force‐generating properties, and various models built using generic human properties. Specifically, the use of *in vivo* individualised muscle force‐generating properties in subject‐specific lower limb musculoskeletal models built from MRI and DTI is investigated by comparing simulated maximal muscle torques to those measured experimentally. Torques from the same models with generic data based on both elderly and young individuals will also be compared to these experimental data, to not only assess the accuracy of generic muscle models but also elucidate exactly how much individualised detail is needed in subject‐specific musculoskeletal models to optimise their accuracy. It is hypothesised that models with subject‐specific muscle force‐generating properties from MRI will simulate muscle torques to a significantly higher degree of accuracy than those with generic data. It is also hypothesised that generic data will show smaller errors in simulated torques in individuals with lower muscle strengths compared to those with higher strengths.

## 2 METHODS

For this study, anatomical and experimental data were gathered from 10 subjects (Table S1; 5 males, 5 females; age – 29 ± 3 years; body mass – 67.9 ± 9 kg; height – 175 ± 7 cm; BMI – 21.9 ± 1.6 kg/m^2^) who signed informed consent prior to participating in the study in accordance with ethical approval from the University of Liverpool's Central University Research Ethics Committee for Physical Interventions (Reference number: 3757).

Here, a similar method to that described previously (Charles *et al*., 2019; Charles *et al*., 2019) is used to estimate subject‐specific muscle architecture data from 31 muscles of the right lower limb from each subject. This involves the use of two MRI sequences, T1‐weighted anatomical turbo spin‐echo (TSE) to estimate muscle volumes and visualise muscle attachment points, and diffusion tensor imaging (DTI) to estimate muscle fibre lengths and pennation angles. The general framework has been validated and described in detail by Charles *et al*. (2019) but is also outlined below.

### 2.1 MR image acquisition

All MR images were obtained using a Siemens 3.0 T Prisma scanner (Siemens) with the following sequence parameters: T1‐weighted anatomical TSE – voxel size 0.4395 × 0.4395 × 6.5 mm^3^; repetition time [TR] – 700 ms; echo time [TE] – 28 ms; number of slices – 36 per segment; number of signal averages (NSA) – 1; diffusion‐weighted single‐shot dual‐refocusing spin‐echo planar – voxel size 2.96 × 2.96 × 6.5 mm^3^; TR/TE – 7900/67 ms; 12 direction diffusion gradients; *b* value – 0 and 400 s/mm^2^; strong fat suppression – spectral attenuated inversion recovery [SPAIR]; number of slices – 36 per segment; NSA – 1; and bandwidth – 2,350 Hz/pixel. All the MR images were acquired from the iliac crest to the plantar surface of the foot, with each subject in a supine position and with the lower limbs in the anatomical position. For each subject, images were acquired in an axial slice orientation and repeated for a total of five to six segments, for a total image acquisition time of ~35 mins. The diffusion tensor images were merged to form a continuous stack during post‐processing using the Stitching plugin for Fiji/ImageJ (Preibisch *et al*., 2009; Schindelin *et al*., 2012).

### 2.2 MR image processing

**FIGURE 1:**
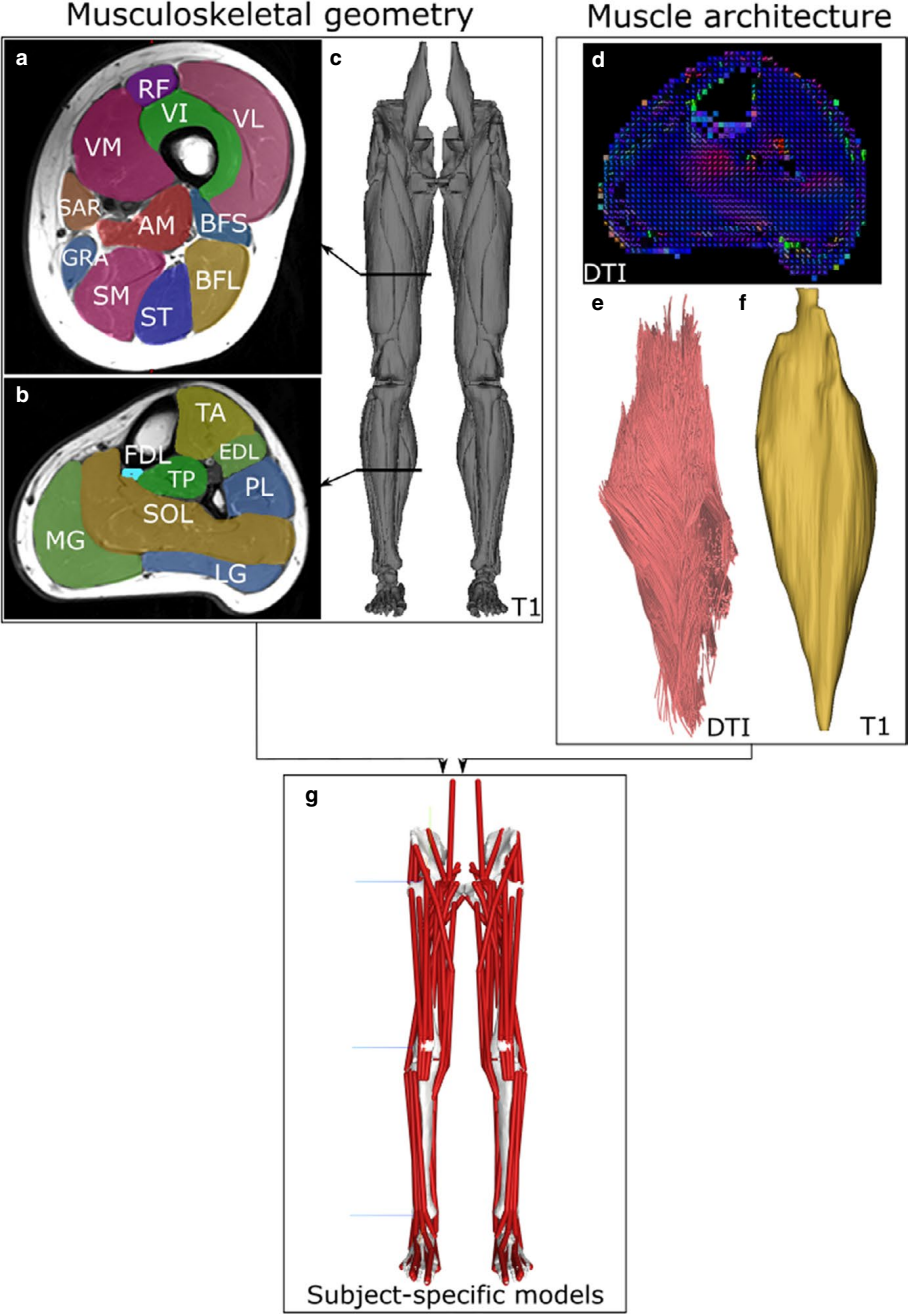
Overview of the subject‐specific musculoskeletal modelling framework. Individualised musculoskeletal geometry (bones and muscle attachment points) for each subject was obtained from digital segmentation of 31 muscles of the lower limb from T1 magnetic resonance images (a–c). Muscle architecture for each muscle was estimated from diffusion tensor imaging (DTI), where fibre orientations can be estimated based on relative water diffusion (d; blue colour represents proximo‐distal fibre orientation, and red represents antero‐posterior orientation). The resulting muscle fibre lengths (e) and muscle volumes (f) were used to calculate maximum isometric force, which were input as force‐generating properties into subject‐specific lower limb musculoskeletal models (g)

The T1‐weighted MR images were digitally segmented in Mimics (Materialise) to create three‐dimensional meshes of each muscle (Figure 1a–c). All DT images were pre‐processed prior to analysis, using similar steps to those outlined in previous studies (see Charles *et al*., (2019); Charles *et al*., (2019) for more details). First, all diffusion images were registered to their respective B0 images using an affine transformation in Dtistudio (Jiang *et al*., 2006) to account for image artefacts and noise as a result of movement during scanning or spatial distortion (e.g. eddy currents and/or magnetic field inhomogeneity). To reduce signal‐to‐noise ratio, the images were then filtered in Medinria (www.med.inria.fr) software using a Rician noise suppression algorithm (Aja‐Fernandez *et al*., 2008). Diffusion tensors were calculated for each voxel using DSI Studio software as explained previously (Bolsterlee *et al*., 2019). Fibre tractography using a deterministic algorithm (Yeh *et al*., 2013) was then carried out in DSI studio software to obtain 5,000 raw fibre tracts, which were assumed here to be functionally equivalent to muscle fascicles and by extension muscle fibres from each muscle of the lower limb (see Charles *et al*., (2019); Charles *et al*., (2019) for a discussion of this assumption). The tracts were created from seed regions of interest (ROIs) placed by overlaying the anatomical T1 images over the DT images and terminated when one of the following stopping criteria was met: step size – 1 mm; fractional anisotropy – >0.5; and angle between segments – >30°. A maximum tract length stopping criteria was also used but was altered for each muscle. The value was set based on muscle belly length values measured from the volumetric muscle meshes obtained from the T1 MR images (assuming that muscle fascicle length cannot exceed muscle length) (Figure 1d–f).

A measurement of muscle fibre length was obtained for each muscle using an ‘anatomically constrained tractography’ post‐processing method previously described (Bolsterlee *et al*., 2019). Here, the raw fibre tracts are constrained in their length based on their corresponding volumetric mesh from the T1 images, which ensures no muscle fibre travels beyond a muscle boundary or is beyond an anatomically realistic length. A value for pennation angle for each muscle was also calculated using this framework.

### 2.3 Calculating muscle force‐generating properties

Estimates of *L*_f_ for each MTU were obtained from:

(1) $$L_{f}=L_{f^{\prime }}\left(\frac{2.7\;\mu m}{L_{s}}\right)$$

where *L*_f_ is optimal fibre length, *L*_f_′ is raw fibre length (measured from DTI), *L*_s_ is sarcomere length, and 2.7 µm is a generic value for optimal sarcomere length (Felder *et al*., 2005). *L*_s_ values were obtained from Ward *et al*. (2009), who measured *L*_s_ in fixed muscles dissected from limbs with most joints (other than the ankle joint) in the anatomical position, as in the present study. Values for *L*_ts_ were estimated from a numerical optimisation algorithm, which estimates this parameter based on MTU and fibre lengths (Manal and Buchanan, 2004). Muscle volumes (*V*_m_) were estimated for each muscle from volumetric muscle meshes from digital segmentation of the T1 MR images.

These parameters were then used to calculate physiological cross‐sectional area (PCSA, mm^2^), a major determinant of muscle force output, using the equation (from Sacks and Roy (1982)):

(2) $$\text{PCSA}=\left(V_{m}*\cos\theta \right)/\left(L_{f}\right)$$

where *V*_m_ is muscle (belly) volume (mm^3^), *L*_f_ is optimal muscle fibre length (mm), and θ is muscle fibre pennation angle. To estimate *F*_max_, individual PCSA values were multiplied by the isometric stress of skeletal muscle (or specific tension; 0.3 N/mm^2^; Zajac (1989)).

### 2.4 Building musculoskeletal models

Subject‐specific musculoskeletal models of both lower limbs were created for each subject in this study using NMSBuilder (Valente *et al*., 2017) (Figure 1g). Each model consisted of 7 bodies (pelvis, right thigh, left thigh, right leg, left leg, right foot and left foot) and 92 musculotendon unit actuators. The mass properties of these bodies (mass, centre of mass and inertia) were estimated from volumetric meshes of each segment created from the T1 MR images. A density of 1,062 kg/m^3^ was assumed for the limb bodies and 1,013 kg/m^3^ for the pelvis body (Dempster and Gaughran, 1967. Joint centres of rotation and body coordinate systems were placed and oriented for the hip, knee and ankle joints based on ISB recommendations (Wu *et al*., 2002). Muscle attachment (origins and insertions) and via points were placed based on the volumetric meshes of the muscles themselves obtained from digital segmentation of the T1 MR images, with each attachment point placed as close to the observed centroid of muscle attachment as possible. Muscles with broad origins were represented by multiple MTU actuators in each model to better recreate their functions. Here, the gluteus maximus, gluteus medius and gluteus minimus muscles were each represented by 3 MTUs, while the adductor magnus muscle was represented by 2 MTUs. To account for this, the calculated *F*_max_ value for these muscles was equally divided between the MTUs, which is a common assumption within musculoskeletal models (Gatesy and English, 1993; Arnold *et al*., 2010; Charles *et al*., 2016). Wrap objects were placed throughout the lower limb in order to ensure each MTU followed a realistic path of action around bones and other MTUs (see Table S3 for general properties of these wrapping objects).

For each subject, five musculoskeletal models were created, each based on the same individualised musculoskeletal geometry created in NMSBuilder. That is, all five models retained the same 3D bone geometry and muscle paths, but varied in *F*_max_, θ, *L*_f_ and *L*_ts_ in the following ways:

- Subject‐specific (SS) – Individualised musculoskeletal geometry and muscle force‐generating properties from MRI and DTI. These properties are listed in Tables S4‐S13.
- Generic elderly (GE) – Individualised musculoskeletal geometry with cadaveric muscle force‐generating properties from literature (Ward *et al*., 2009) (age – 89 ± 3 years; body mass – 82.7 ± 15.3 kg), which has been used to wholly or partially inform various iterations of generic musculoskeletal models within the SIMM or OpenSim frameworks (Delp *et al*., 1990; Delp and Loan, 2000; Thelen *et al*., 2003; Arnold *et al*., 2010; Rajagopal *et al*., 2016).
- Generic elderly optimised (GE_O_) – Individualised musculoskeletal geometry with pennation angle values from cadaveric studies and optimised *L*_f_ and *L*_ts_ obtained using a muscle optimiser algorithm (Modenese *et al*., 2016). This optimises these values based on a validated reference musculoskeletal model (Rajagopal *et al*., 2016) and the anthropometry of the target (subject‐specific) model geometry, and therefore ensures the MTUs operate on the correct part of their force–length curve. *F*_max_ values were scaled to each individual based on using the following formula from van der Krogt et al. (van der Krogt *et al*., 2016):

(3) $$F_{\max}^{\text{scaled}}=F_{\max}^{\text{generic}}\times {\left(\frac{M^{\text{subject}}}{M^{\text{generic}}}\right)}^{\left(2/3\right)}$$

where M^subject^ is the body mass of each subject in this study, and M^generic^ is the body mass of a standard generic model, taken here as 75.3 kg (from Rajagopal *et al*. (2016)).

**FIGURE 2:**
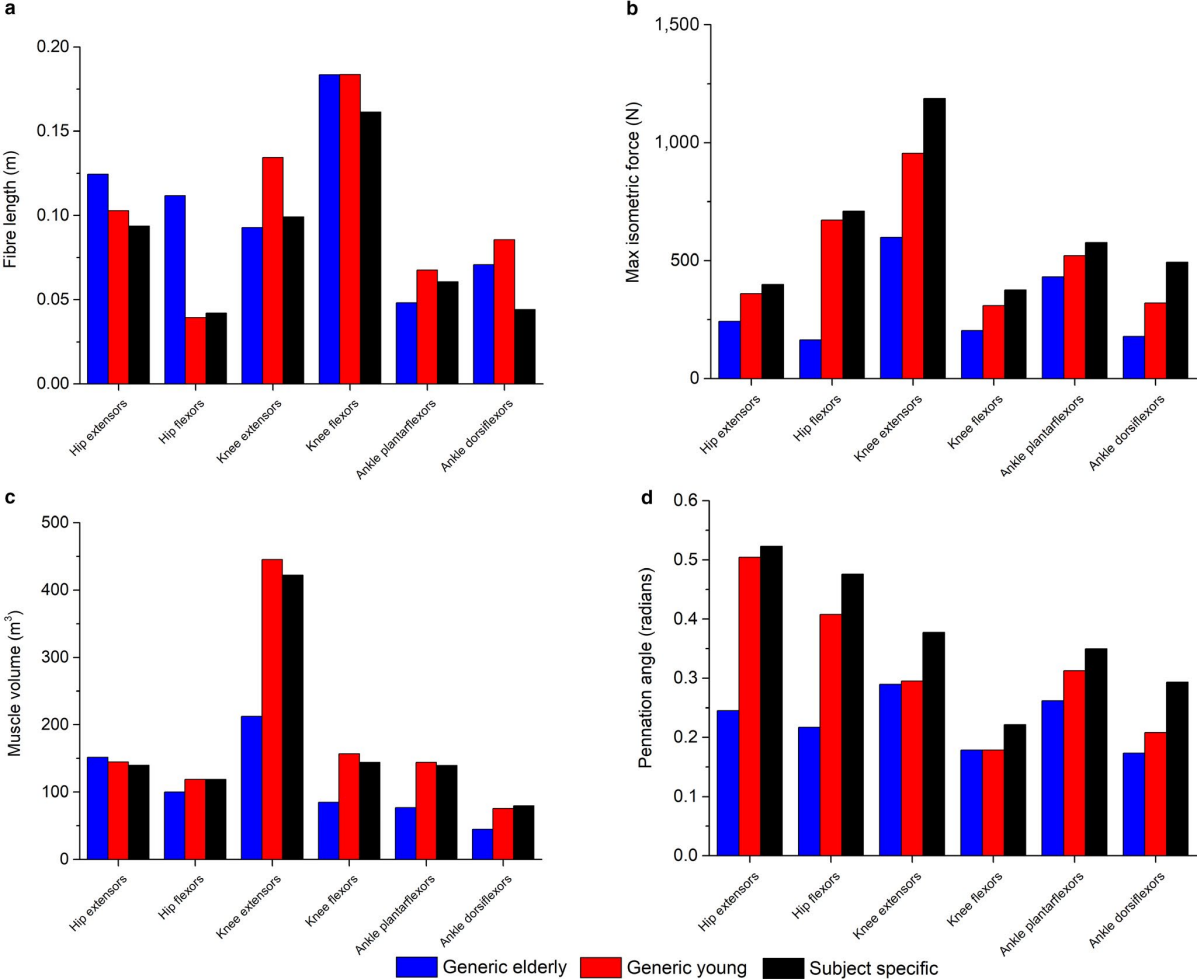
Functional group mean scaled maximum isometric force values (a), muscle fibre lengths (b) and pennation angles (c) from an elderly generic anatomical data set (Ward *et al*., 2009), a young generic data set collected from MRI (a combination of data collected here and previously (Charles *et al*., 2019)) and subject‐specific data (averaged data collected here). For muscle functional groupings, see Table S1

- Generic young (GY) – Individualised musculoskeletal geometry with muscle force‐generating properties from young, healthy individuals (age – 28 ± 4 years; body mass – 71.9 ± 11 kg). This is a combined average of the data collected in the present study and a previously published in vivo data set collected using similar methods (Charles *et al*., 2019). Relative to the elderly generic data, the muscles within this data set are characterised by higher muscle volumes and *F*_max_ values, longer fibre lengths (particularly in more distal functional groups) and larger pennation angles (Figure 2).
- Generic young optimised (GY_O_) – Individualised musculoskeletal geometry with pennation angle values from young, healthy individuals, and optimised *L*_f_ and *L*_ts_ values obtained from the same algorithm as the GE_O_ models. *F*_max_ values were scaled using equation (3), but with M^generic^ in this case set as 71.9 kg (the mean body mass of the individuals used to generate that anatomical data set).

### 2.5 Isokinetic and isometric torque measurement

**FIGURE 3:**
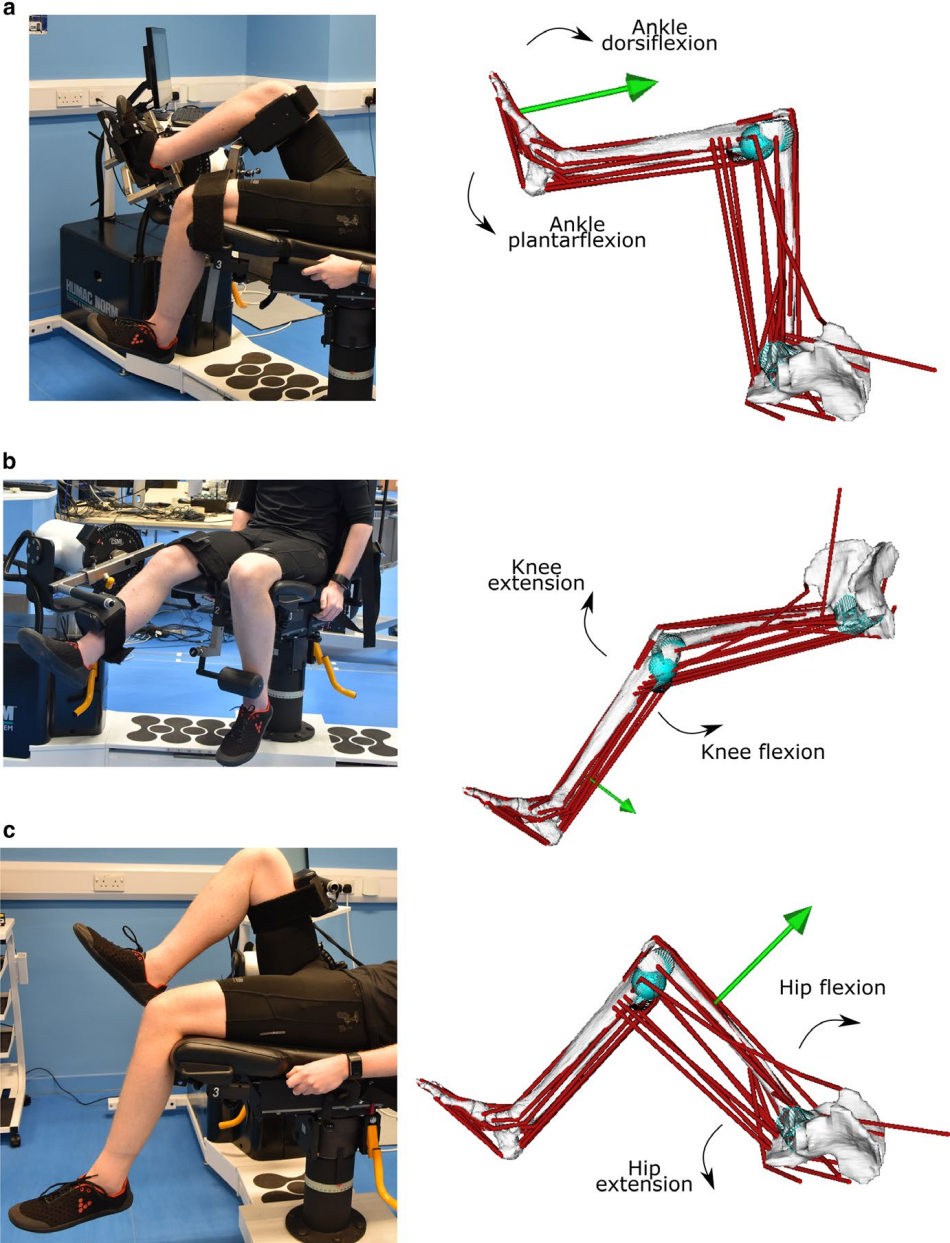
The experimental and simulation procedures, where isokinetic trials on an isokinetic dynamometer through ankle plantarflexion–dorsiflexion (a), knee extension–flexion (b) and hip extension–flexion (c) are replicated using subject‐specific musculoskeletal models within OpenSim to estimate the abilities of the musculotendon units to produce the desired motion. The green arrows represent the external forces applied to each model through each motion, which are derived from the experimentally derived muscle torques measured from the isokinetic dynamometer

To provide an experimental, functional metric against which to validate the musculoskeletal models, an isokinetic dynamometer (HUMAC NORM, CSMi) was used to measure right maximal lower limb muscle torques from each subject. Torques were measured through ankle plantarflexion–dorsiflexion, knee extension–flexion and hip extension–flexion during both isokinetic and isometric conditions. For each of these rotations, each subject's maximum flexion and extension angles were measured to ensure each isokinetic trial measured muscle torques throughout their entire respective ranges of motion (average joint angles are reported in Table S14). For each isokinetic trial, each subject was restrained in a way to allow only the joint of interest to rotate, in accordance with CSMi guidelines (see Figure 3). Each isokinetic trial was composed of 5 repetitions, while each isometric repetition lasted 5 s with 5‐s rest between each repetition, for a total of 5 repetitions (see Table S14 for more information). Each subject was given verbal encouragement during each trial to ensure they exerted maximum torque.

### 2.6 Simulation procedure

For each subject, one representative isokinetic repetition from each joint was simulated in the SS, GE, GE_O_, GY and GY_O_ models within OpenSim 4.0 (Seth *et al*., 2018). To do so, the measured isokinetic torques throughout each movement from each subject were converted to an external force and applied to the models at the appropriate point (ankle plantarflexion/dorsiflexion – the metatarsophalangeal joint; knee extension/flexion – the distal tibial shaft; hip extension/flexion – the distal femoral shaft; see Figure 3). With these external forces applied, static optimisation was used to predict a set of individual MTU forces and activations which could satisfy the applied loads, with the objective function of minimising squared muscle activations. To facilitate a direct comparison to the experimentally measured torques (*T*_e_) from the isometric dynamometer, the relevant predicted muscle forces from each simulation were multiplied by their respective moment arms and then summed to obtain a value for total predicted muscle torque (*T*_p_). In a wholly accurate model and simulation, *T*_p_ would be equal to *T*_e_, as the MTUs within the model would be able to produce enough force to satisfy the external loads and perfectly simulate in vivo muscle torques measured by the isokinetic dynamometer. If the models are unable to satisfy the external loads, the MTUs will reach maximal activation and be unable to produce enough force to produce the desired motion, resulting in a failed static optimisation. No reserve actuators were applied to the static optimisation simulations, in order to ensure that these analyses were focusing only on the functional capabilities of the MTUs within the models (see Appendix S1 for details and outputs of simulations performed with reserve actuators appended to each model).

For each simulation, root‐mean‐squared errors (RMSE) of *T*_p_ relative to *T*_e_ were calculated to quantify the agreement between the two data sets in SS, GE, GE_O_, GY and GY_O_ models: [Correction added on 2 July 2020, after first online publication: Equation 4 has been amended.]

(4) $$\text{RMSE =}\;\sqrt{\bar{{\left(T_{e}-T_{p}\right)}^{2}}}$$

A one‐way ANOVA with Tukey’s post hoc comparisons was performed to test for statistically significant differences between the RMSE values obtained from the models of each subject. These statistical analyses were performed in OriginPro software (version 2016. OriginLab Corporation).

The linear regression function in OriginPro software was used to test for correlations between experimentally derived maximum isometric torque (*T*_max_; from isometric trials on the isokinetic dynamometer) values from each subject and individual RMSE values for each isokinetic trial in SS, GE, GE_O_, GY and GY_O_ models. This was used to test the hypothesis that generic data will show smaller errors in simulated torques in individuals with lower muscle strengths compared to those with higher strengths. Here, *T*_max_ values were assumed to be representative of an individual's maximal muscle strength. For example, a strong positive correlation between *T*_max_ and RMSE through a particular joint rotation within a particular model type would mean that muscle torques in stronger individuals are predicted by the simulation less accurately than those in weaker individuals, thus supporting this hypothesis.

In order to assess the ability of each type of model to predict inter‐subject variations in muscle outputs, Spearman's rank correlation coefficient was calculated (IBM SPSS Statistics for Windows, version 25.0. Armonk, NY: IBM Corp) for the predicted maximum isokinetic muscle torques through ankle plantarflexion, knee extension and hip extension from each model for each subject relative to their experimentally measured isokinetic muscle torques from the isokinetic dynamometer (*p* < 0.05). This test was used to give an indication of how well each model predicts the ranked order of subjects in terms of measured maximum isokinetic torque and therefore potentially how well each type of model is able to reflect inter‐subject variations in muscle forces and functions.

## 3 RESULTS

### 3.1 RMS errors

**FIGURE 4:**
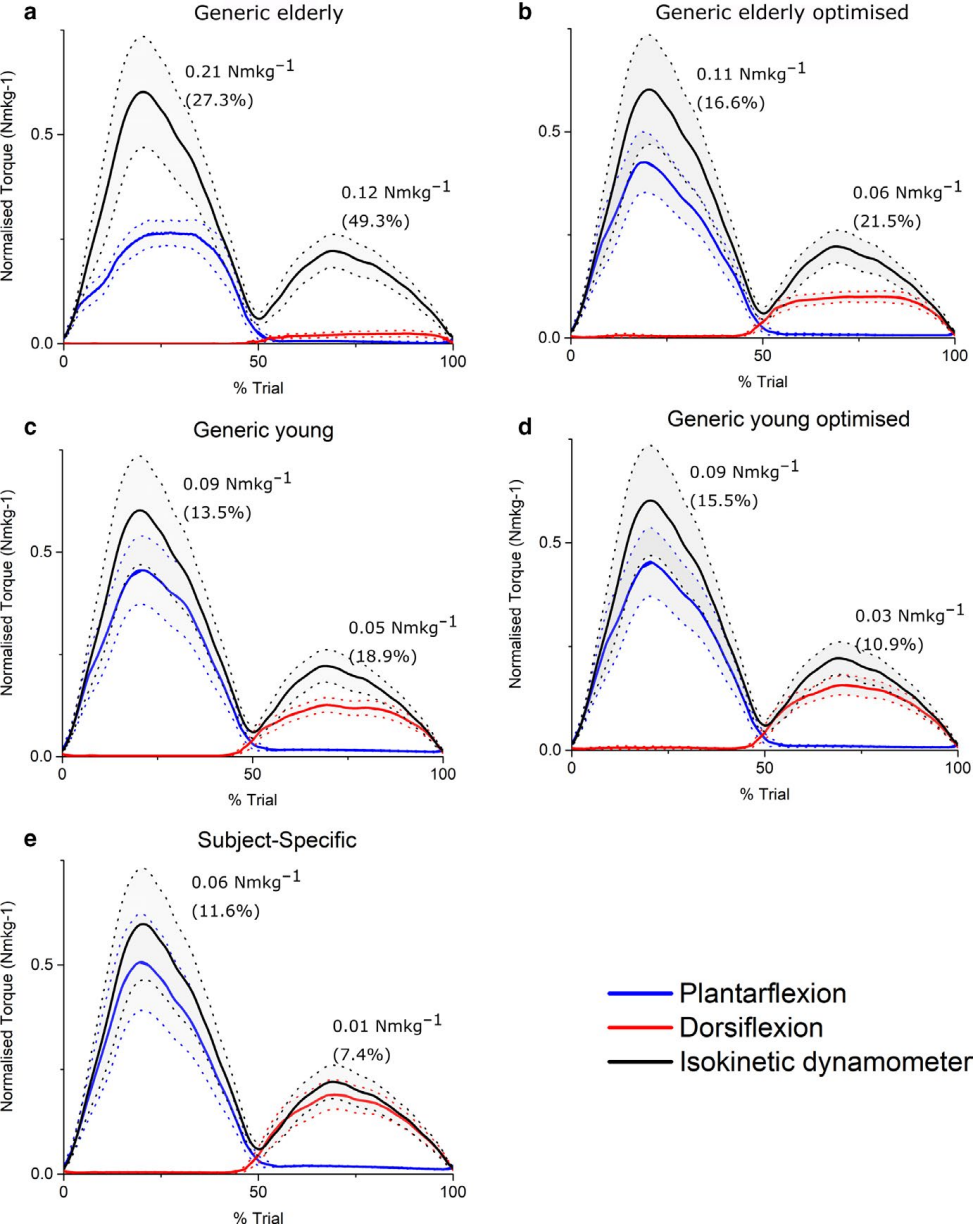
Mean (±standard error) normalised predicted ankle muscle torques from static optimisation in the Generic elderly (a), Generic elderly optimised (b), Generic young (c), Generic young optimised (d) and Subject‐specific (e) models against mean experimentally derived normalised ankle muscle torques measured from an isokinetic dynamometer. Root‐mean‐squared errors expressed in Nm/kg and as % of maximum isokinetic torque show that on average, the subject‐specific models predicted maximal muscle torques to the greatest degree of accuracy through both ankle plantarflexion and dorsiflexion relative to all other models

**FIGURE 5:**
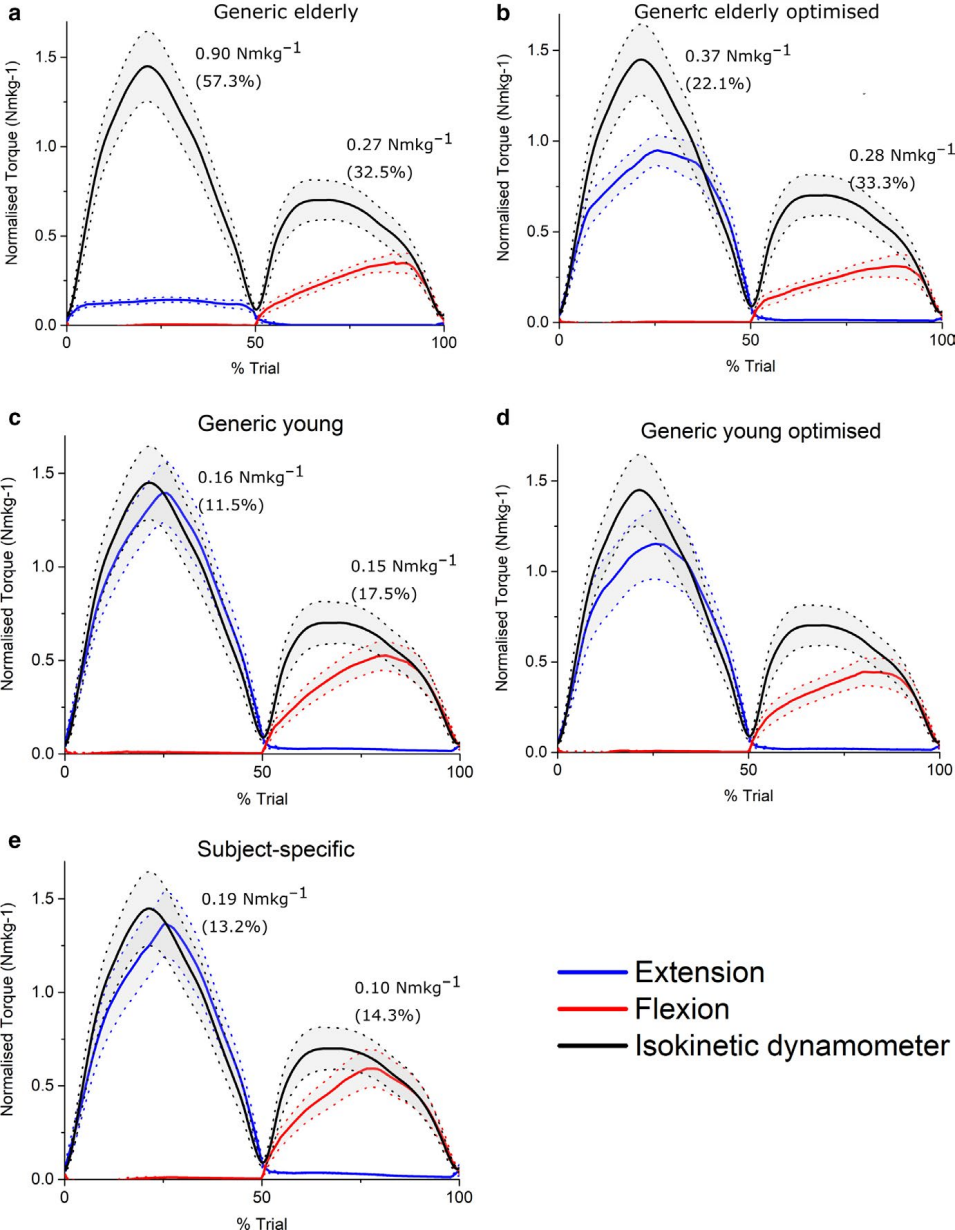
Mean (±standard error) normalised predicted knee muscle torques from static optimisation in the Generic elderly (a), Generic elderly optimised (b), Generic young (c), Generic young optimised (d) and Subject‐specific (e) models against mean experimentally derived normalised knee muscle torques measured from an isokinetic dynamometer. Root‐mean‐squared errors, expressed in Nm/kg and as % of maximum isokinetic torque, show that the generic young models predicted maximal muscle torques to the greatest degree of accuracy through knee extension, while the subject‐specific models were the most accurate through knee flexion

**FIGURE 6:**
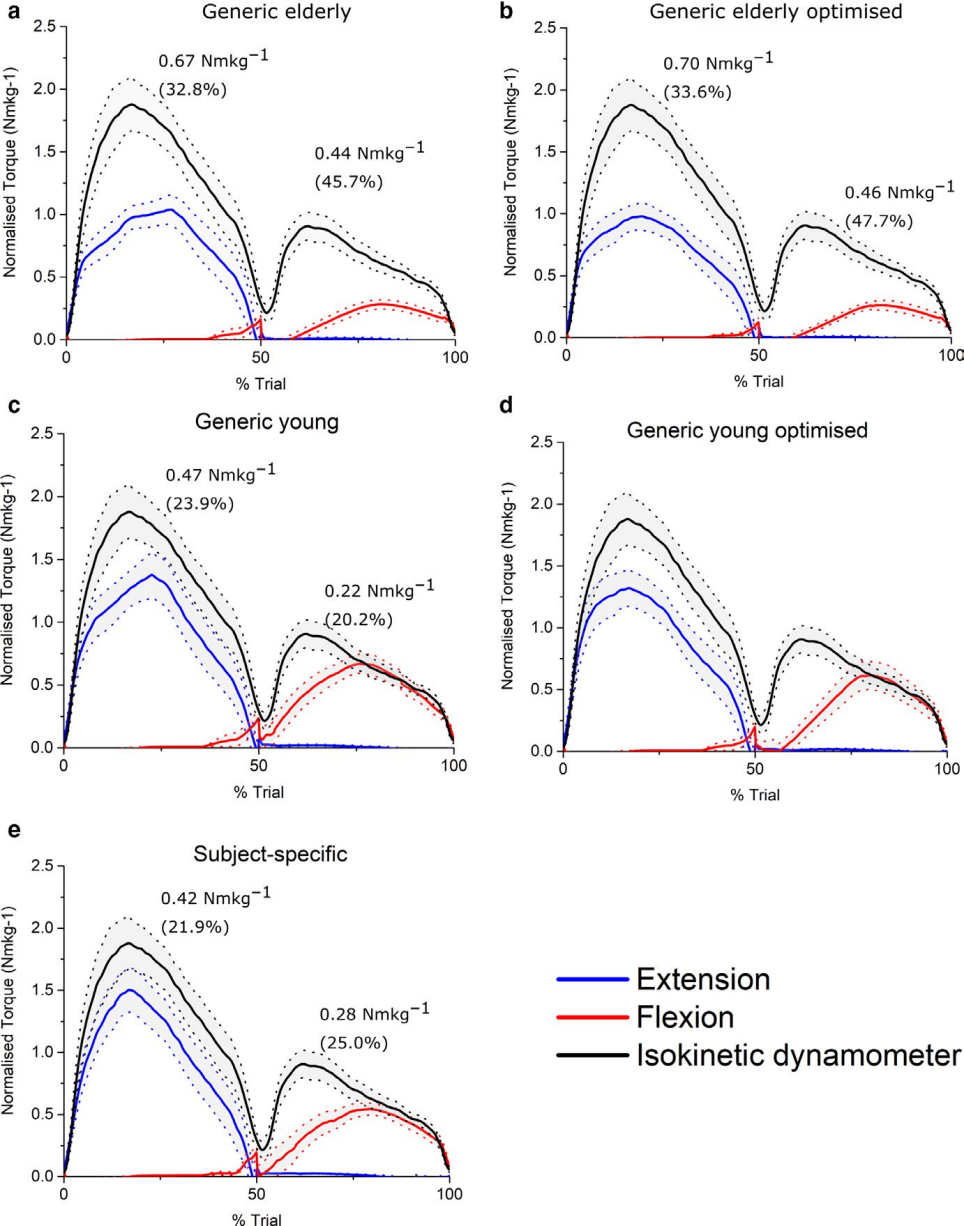
Mean (±standard error) normalised predicted hip muscle torques from static optimisation in the Generic elderly (a), Generic elderly optimised (b), Generic young (c), Generic young optimised (d) and Subject‐specific (c) models against mean experimentally derived normalised hip muscle torques measured from an isokinetic dynamometer. Root‐mean‐squared errors, expressed in Nm/kg and as % of maximum isokinetic torque, show that the subject‐specific models predicted maximal muscle torques to the greatest degree of accuracy through hip extension, but the generic young models were the most accurate through hip flexion

**FIGURE 7:**
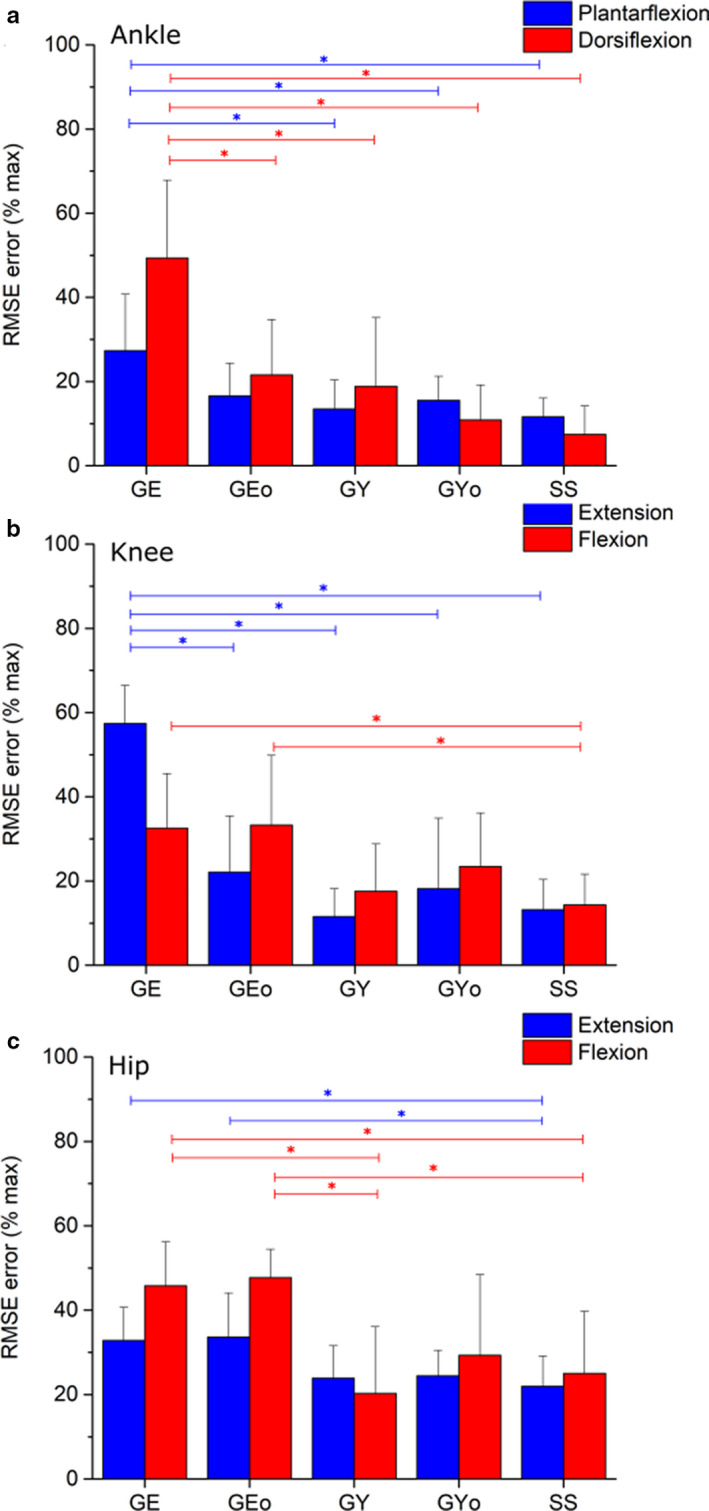
Average root‐mean‐squared errors (expressed as % of maximum isokinetic torque, +1 standard deviation) of simulated muscle torques relative to experimentally measured ankle (a), knee (b) and hip (c) torques from an isokinetic dynamometer. *Indicates statistically significant differences in mean RMS errors as predicted by the ANOVA (*p* < 0.05). Through ankle plantarflexion and dorsiflexion, as well as knee extension, the Generic elderly (GE) models showed significantly larger RMSEs than the Generic young (GY), Generic young optimised (GY_O_) and Subject‐specific (SS) models. Through knee flexion, hip extension and hip flexion, the SS models had significantly lower RMSEs than both the GE and Generic elderly optimised (GE_O_) models. Also through hip flexion, the GE models had significantly higher RMSEs than the GY models, while the GE_O_ models had significantly higher RMSEs than the GY, Generic young optimised GY_O_ and SS models. For specific *p* values, see Table S17

At the ankle, knee and hip joints, the fully subject‐specific musculoskeletal models predicted muscle torques to a greater average accuracy than all other models (Figures 4, 5, 6, 7). On average, the SS models predicted ankle plantarflexion and ankle dorsiflexion muscle torques with RMS errors of 11.6% (% of maximum *T*_e_) and 7.4%, respectively (Figure 4e), while the GE and GE_O_ models showed errors of 27.3% and 16.6% through plantarflexion, and 49.3% and 21.5% through dorsiflexion, respectively (Figure 4a, b). The errors in the GE models through ankle plantarflexion and dorsiflexion were statistically significantly higher than those in the GY, GY_O_ and SS models on average (*p* < 0.05); Figure 7). The errors in the GY and GY_O_ models were closer to those in SS models, with RMS errors of 13.5% and 15.5% through plantarflexion and 18.9% and 10.9% through dorsiflexion, respectively (Figure 4c, d). Knee extension and knee flexion muscle torques were predicted less well than ankle muscle torques by the SS models on average (RMSE – 13.2% and 14.3%), but nevertheless more accurately than both the GE and GE_O_ models (Figure 5a, b, e), where errors ranged from 57.3% to 22.1% through extension and 32.5% to 33.3% through knee flexion. The best agreement to the experimental data through knee extension on average was in the GY models (11.5%) (Figure 5c), but through knee flexion the errors were slightly larger than the SS models (17.5%). Both knee extension and flexion errors were higher in the GY_O_ models than the SS models (18.1% and 23.4%) (Figure 5d). Indeed, only the SS models had significantly lower errors than the GE and GE_O_ models through knee flexion (*p* = 0.02 and 0.01, respectively), while all other models had significantly lower errors than the GE models through knee extension (Figure 7). The highest average errors in SS models were seen through hip flexion and extension, with errors of 21.9% and 25% (Figure 6f). However, these errors were smaller than those seen in the GE and GE_O_ models, which showed errors of 32.8% and 33.6% during hip extension and 45.7% and 47.7% during hip flexion (Figure 6a, b). Both GY and GY_O_ models had larger errors than the SS models through hip extension (23.9% and 24.4%), but the errors through hip flexion were smaller in the GY models on average (20.2%) (Figure 6c, d). Only the SS models had significantly smaller errors than the GE models through hip extension (*p* = 0.04), while the SS and GY models had significantly smaller errors than the GE models through hip flexion (*p* = 0.02 and 0.002, respectively). The GY and SS models all showed significantly smaller errors than the GE_O_ models through hip flexion on average (*p* = 0.001 and 0.01, respectively; Figure 7).

Between all the subjects, the smallest RMS errors in the SS models were seen in Subject 5 through ankle dorsiflexion (0.05% of max *T*_e_), while the largest errors were seen in Subject 6 through hip flexion (44.5%). In the GE models, the largest errors were generally seen in knee extension, with Subject 4 having the largest at 65.6%, and the same individual also had the smallest error through ankle plantarflexion at 9.14%. Subject 10 had the smallest error of the GE_O_ models, with an RMS error of 0.27% through ankle dorsiflexion, while Subject 7 showed the largest error in these models (58% through hip flexion). Similar to the SS models, the smallest error in the GY models was seen in Subject 5 through ankle dorsiflexion (0.94%), but the largest error was seen in Subject 4 through hip flexion (46.3%). Subject 3 had the smallest error of the GYo models, with an error of 1.30% through ankle dorsiflexion, while Subject 7 had the largest error with 67.1% through hip flexion. See Tables S15 and S16 for individual RMSE values for all models of each subject in this study, and see Table S17 for the statistical significance (*p* values) of the differences between these RMSE values within each model as predicted by the ANOVA.

### 3.2 Linear regression

**FIGURE 8:**
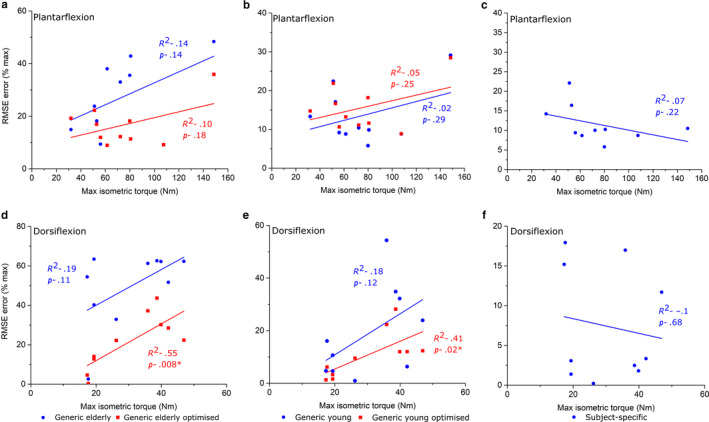
Linear regression between experimentally derived maximum isometric ankle muscle torque measured from an isokinetic dynamometer and root‐mean‐squared errors of predicted isometric muscle torques in Generic elderly, Generic elderly optimised, Generic young, Generic young optimised and Subject‐specific models through ankle plantarflexion (a–c) and ankle dorsiflexion (d–f). Adjusted *R* ^2^ values are shown alongside associated *p* values. *Statistical significance (*p* < 0.05). The statistically significant positive correlations seen in the Generic elderly optimised and the Generic young optimised models through ankle dorsiflexion suggest that there is a trend for these models to accurately predict muscle torques within individuals with low muscle force capabilities, but these muscle data may not be suitable for simulating muscle functions in stronger individuals

**FIGURE 9:**
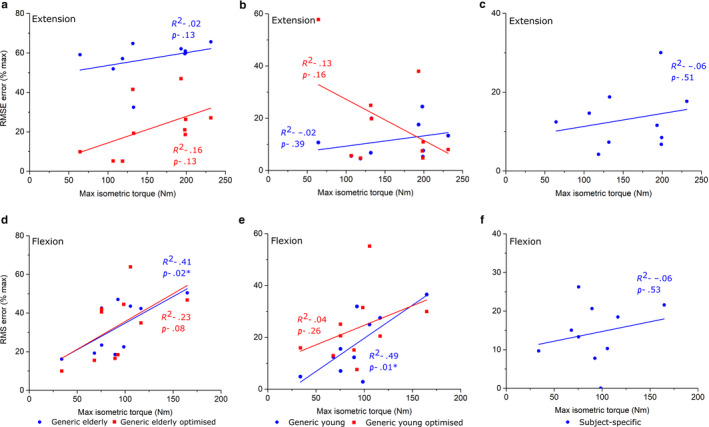
Linear regression between experimentally derived maximum isometric knee muscle torque measured from an isokinetic dynamometer and root‐mean‐squared errors of predicted isometric muscle torques in Generic elderly, Generic elderly optimised, Generic young, Generic young optimised and Subject‐specific models through knee extension (a–c) and knee flexion (d–f). Adjusted *R* ^2^ values are shown alongside associated *p* values. *Statistical significance (*p* < 0.05). The statistically significant positive correlations seen in the Generic elderly, Generic elderly optimised and the Generic young models through knee flexion suggest that there is a trend for these models to accurately predict muscle torques within individuals with low muscle force capabilities, but these muscle data may not be suitable for simulating muscle functions stronger in individuals

**FIGURE 10:**
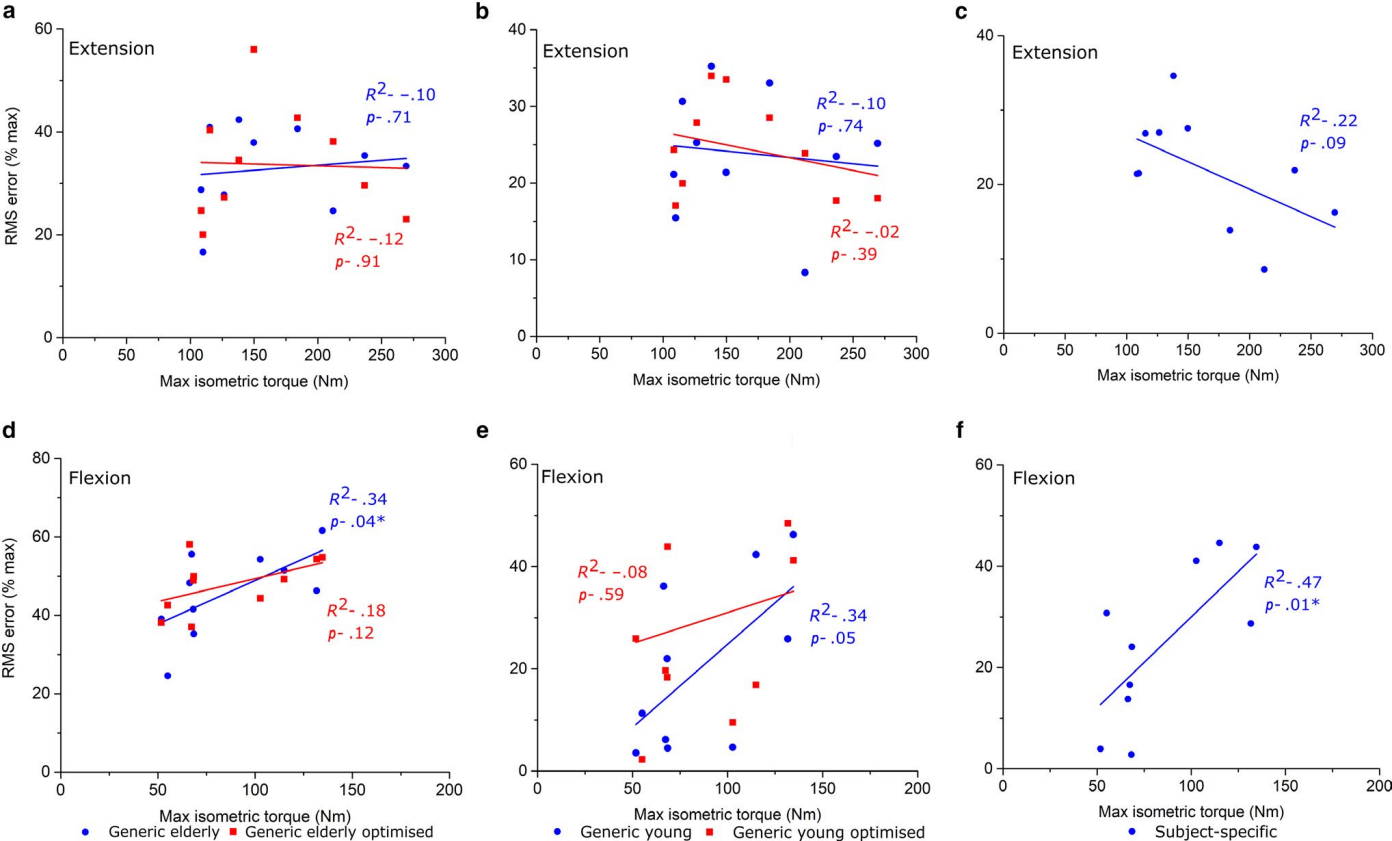
Linear regression between experimentally derived maximum isometric hip muscle torque measured from an isokinetic dynamometer and root‐mean‐squared errors of predicted isometric muscle torques in Generic elderly, Generic elderly optimised, Generic young, Generic young Optimised and Subject‐specific models through hip extension (a–c) and hip flexion (d–f). Adjusted *R* ^2^ values are shown alongside associated *p* values. *Statistical significance (*p* < 0.05). The statistically significant positive correlations seen in the Generic elderly models through hip flexion suggest that there is a trend for these models to accurately predict muscle torques within individuals with low muscle force capabilities, but these muscle data may not be suitable for simulating muscle functions in stronger individuals. The statistically positive correlations and high root‐mean‐squared errors in the subject‐specific models through hip flexion suggest the framework of measuring muscle architecture from magnetic resonance images could underestimate the force‐generating capacities of these muscles, a deficiency which is exacerbated in models of stronger individuals

The strongest correlations between *T*_max_ and individual RMSE were generally seen in both the elderly generic models through ankle plantarflexion/dorsiflexion (Figure 8a, d) and knee extension/flexion (Figure 9a, d). Statistically significant positive correlations were found during ankle dorsiflexion (*R* ^2^ = 0.55) in the GE_O_ models and during knee flexion and hip flexion in the GE models (*R* ^2^ = 0.42 and 0.34, respectively) (Figures 9a, 10a). In the GY models, only knee flexion *T*_max_ was significantly positively correlated with RMSE (*R* ^2^ = 0.49) (Figure 9e), while in the GY_O_ models, only ankle dorsiflexion *T*_max_ was significantly correlated with RMSE (*R* ^2^ = 0.41) (Figure 8e). Only through hip flexion were RMSE and *T*_max_ significantly correlated in the SS models (Figure 10f).

### 3.3 Spearman's rank correlation coefficients

The maximum predicted isokinetic torques in the SS models had the largest Spearman's rank correlation coefficients (Table 1) relative to the measured isokinetic torques through all three movements tested (ankle plantarflexion – *ρ* = 0.988; knee extension – *ρ* = 0.976; hip extension – *ρ* = 0.927). The GY and GY_O_ models also had high coefficients at all joints (*ρ* = 0.988 and *ρ* = 0.939 through ankle plantarflexion, *ρ* = 0.952 and *ρ* = 0.939 through knee extension and *ρ* = 0.903 and *ρ* = 0.915 through hip extension), while the GE and GE_O_ models had the lowest coefficients (*ρ* = 0.612 and *ρ* = 0.952 through ankle plantarflexion, *ρ* = −0.442 and *ρ* = 0.806 through knee extension and *ρ* = 0.867 and *ρ* = 0.758 through hip extension).

**Table 1:** Spearman's rank correlation coefficients (*ρ*) when comparing ranked maximum isokinetic torques predicted by each model to those measured experimentally by an isokinetic dynamometer through ankle plantarflexion, knee extension and hip extension

Maximum isokinetic torque	Model	Spearman's *ρ*
Ankle plantarflexion	SS	0.988*
	GE	0.612
	GE_O_	0.952*
	GY	0.988*
	GY_O_	0.939*
Knee extension	SS	0.976*
	GE	−0.442
	GE_O_	0.806*
	GY	0.952*
	GY_O_	0.939*
Hip extension	SS	0.927*
	GE	0.867*
	GE_O_	0.758*
	GY	0.903*
	GY_O_	0.915*

Abbreviations: GE, Generic elderly; GEo, Generic elderly optimised; GY, Generic young; GYo, Generic young optimised; SS, Subject‐specific.

* Statistical significance (*p* < 0.05).

## 4 DISCUSSION

This study aimed to quantify the absolute and relative ability of subject‐specific versus generic muscle architecture data to predict MTU forces and torques within musculoskeletal models. Torques were predicted using OpenSim within subject‐specific skeletal models with subject‐specific, generic and generic optimised muscle force‐generating properties (from both elderly and young individuals), and directly compared to experimentally derived isokinetic muscle torques from an isokinetic dynamometer.

These results provide relatively strong validation of the presented subject‐specific musculoskeletal modelling framework, with the SS models (containing individualised muscle force‐generating properties) matching the experimental data significantly better than their equivalent GE and GE_O_ models at all joints tested. The difference to the GY and GY_O_ models was smaller in terms of root‐mean‐squared errors; however, for most of the movements analysed, the SS models predicted muscle torques to a greater degree of accuracy (Figures 4, 5, 6, 7). This supports the initial hypothesis that musculoskeletal models containing subject‐specific muscle architecture and musculoskeletal geometry would simulate muscle torques to a greater degree of accuracy than those containing generic muscle force‐generating properties. The failure of the elderly generic‐based models to accurately simulate muscle torques, even with optimised muscle fibre lengths, was not surprising and reflects a shift away from exclusively using such data in recent biomechanical modelling studies with the increased availability of MRI‐based anatomical data sets (Handsfield *et al*., 2014; Charles *et al*., 2019). Nevertheless, the results obtained here highlight and support some potentially interesting functional effects of known changes in muscle architecture which occurs due to ageing. The largest RMS errors in these models were during knee extension (57% of maximum isometric torque), and it is within these muscles that decreases in muscle torque with age are the most apparent (Moore *et al*. (2014), see also Figure 3). While several factors have been proposed to be the cause of this functional decline in muscle quality with age, such as decreases in motor unit activation and muscle fibre‐specific tension (Narici *et al*., 1985; Narici *et al*., 2008), the results here suggest that muscle architecture could have a significant role.

Regarding the models based on muscle and fibre architecture data from young, healthy individuals, the relatively small RMSE values in the SS, GY and GY_O_ models (Figures 4, 5, 6, 7) suggest that such data obtained from MRI accurately reflect in vivo muscle force‐generating capacities (on a muscle group level) and therefore could accurately simulate muscle function in a computational context. This supports the use of these and similar medical imaging‐based anatomical data in musculoskeletal models, particularly when simulating maximal effort tasks in young, healthy individuals. However, these results suggest that scaling these young generic data has little impact on their potential accuracy. Scaling or optimising muscle architecture data to specific individuals based on relative body mass or anthropometry is an often used method of estimating personalised force‐generating properties (Correa and Pandy, 2011; van der Krogt *et al*., 2016; Kainz *et al*., 2018). However, these adjusted properties, even those from young generic data, still appear to be less accurate than subject‐specific data from MRI in predicting muscle torques and only provide significant improvements in accuracy within some muscle groups throughout the tested movements. In fact, while optimising elderly generic data improved the accuracy of the cadaver‐derived muscle force‐generating properties (statistically significantly through ankle dorsiflexion), optimising the young generic properties on average had little effect on resulting RMS errors and even increased them through all movements other than ankle dorsiflexion (Figures 4, 5, 6, 7). Given the similar ages and body masses of the individuals in this study and in the young generic anatomical data set, the lack of change in errors after scaling was somewhat expected. Therefore, this should not necessarily discount the use of scaling, optimisation or regression methods to estimate individuals’ muscle force‐generating properties for musculoskeletal models if methods to obtain subject‐specific data are unavailable.

However, while the use of using average muscle force‐generating properties from young, healthy individuals appears to be valid way of simulating muscle function in individuals of a similar age demographic, correlations between individual RMS error and maximum isometric joint torque (indicative of an individual's overall strength) (Figures 7, 8, 9) suggest that this may be subject‐dependent. Significant correlations were seen through ankle dorsiflexion and knee flexion in the GY and GY_O_ models, respectively (Figures 8 and 9), suggesting that for individuals with low strengths around those joints, young generic data can accurately simulate muscle functions, but not for stronger individuals. These correlations were stronger in the elderly generic models, where individual RMSE values showed stronger correlations with *T*_max_ through knee flexion, ankle plantarflexion and ankle dorsiflexion in the GE_O_ relative to the GE models. The second hypothesis that generic data will show smaller errors in simulated torques in individuals with lower muscle strengths compared to those with higher strengths is supported by these results and suggests that generic properties may be applicable for models of individuals with lower muscle force outputs, but not those with higher torque‐generating capacities. Indeed, RMS errors <5% of T_e_ can be seen in the GE_O_ models of S07, S09 and S10 during ankle dorsiflexion and S07 during knee flexion (Tables S15‐S16). This has potential implications for musculoskeletal modelling in a variety of contexts, such as in clinical studies where gait dynamics of pathological populations are investigated using biomechanical simulations (e.g. cerebral palsy (Rosenberg and Steele, 2017)). Such individuals are generally characterised by a reduction in muscle force‐generating capacity relative to younger individuals (D'Souza *et al*., 2019), similar to the elderly generic data set used here. Therefore, if gathering in vivo muscle data from MRI is not possible in these situations, optimising elderly generic muscle properties (Modenese *et al*., 2016) may be sufficient to investigate muscle functions in these individuals using musculoskeletal models. Similarly, when creating animal models, it can be difficult or impossible to obtain extensive muscle force‐generating properties from the specific individual being modelled. The results presented here suggest that including these data from matched individuals (collected as primary data or from published data sets if available) or data from species of similar morphometry or ecologies may provide a suitable solution for predicting muscle functions if obtaining individualised muscle data is not possible.

Nevertheless, these results show that including muscle architecture data from MRI in subject‐specific musculoskeletal models significantly improves accuracy regarding predictions and muscle torques, forces and functions. The data suggest this becomes increasingly important in stronger individuals, and overall, the results indicate that the use of generic musculoskeletal models to assess high‐performance activities in young individuals (particularly in sports biomechanics) is not optimal. This is particularly apparent when considering the inter‐subject variations in predictions of muscle group outputs, as shown by Spearman's rank correlation coefficients for each model in predicted maximum isokinetic torque relative to measured values from the isokinetic dynamometer (Table 1), where the subject‐specific models showed the highest coefficients of all the models through all movements tested. So, while the young generic‐based models predicted muscle torques in general to a similar degree of accuracy than the subject‐specific models when averaged over all subjects, they were not as consistent at doing so between all individuals. These results therefore support the notion that creating individualised models should be considered the optimal approach to investigate how small‐scale variations in musculoskeletal geometry or muscle architecture between individuals influence musculoskeletal function, on a muscle group level at least, which is an exciting but as yet not fully explored benefit of subject‐specific musculoskeletal modelling.

## 5 FUTURE PERSPECTIVES

While this study goes a long way to solidifying the presented framework of subject‐specific musculoskeletal modelling as a valid method of obtaining predictions of muscle functions to a high degree of accuracy, some factors may require greater consideration in future work. For example, while the subject‐specific models showed good agreement with the experimental data relative to the generic models in all 3 of the movements tested, these were purely sagittal plane motions involving rotations through only one degree of freedom (DOF). It is possible that simulating more complex movements (such as gait), where more rotational and translational DOFs are included, would introduce larger degrees of error into the model predictions. However, as experimental data against which to validate models during such movements are difficult to obtain, quantifying this error and validating such complex and dynamic simulations are a challenge and a limitation of many musculoskeletal modelling studies. Comparing predicted muscle activations against experimentally obtained muscle activity from electromyography (EMG) is the most commonly used validation method (Glitsch and Baumann, 1997; Lenaerts *et al*., 2008; Lund *et al*., 2012), and could be useful here to confirm that the models and simulations are truly replicating individual in vivo muscle function, rather than simply finding a different solution to satisfy the applied external forces. However, the functional redundancy within the vertebrate musculoskeletal system (Crowninshield and Brand, 1981; Modenese *et al*., 2013; Simpson *et al*., 2015), which results in many different muscle activation pattern being able to produce the same movement, is an inherent drawback of this validation method and means agreements between predicted muscle activations and measured muscle activity can be difficult to obtain and qualify (see Lund *et al*. (2012) for further discussion on the limitations of EMG validation). So, while these results do not necessarily confirm that models constructed with subject‐specific data predict individual muscle torques, forces and functions to a higher degree of accuracy than other models, the relatively low RMS errors through all movements tested suggest they have the greatest potential to do so. Validating predictions from these models through simulations of more complex movements such as gait against experimental data in future studies will help to confirm these assertations.

Additionally, while the framework of including muscle architecture‐derived MRI and DTI in subject‐specific models presented here represents a novel contribution to the subject‐specific modelling field, improvements could still be made to increase the level of individualisation. This is particularly apparent in the estimation of optimal fibre lengths and tendon slack lengths which, as previously discussed, are parameters to which musculoskeletal models are highly sensitive (Scovil and Ronsky, 2006; Redl *et al*., 2007; Ackland *et al*., 2012; Charles *et al*., 2016). Optimal fibre lengths were estimated by normalising the DTI‐derived muscle fibres to sarcomere lengths from previous literature (Ward *et al*., 2009), and tendon slack lengths were estimated using a numerical optimisation algorithm (Manal and Buchanan, 2004). Both methods rely on generic muscle data or assumptions and therefore limit the subject specificity of the force–length relationships in each muscle. However, given the current difficulties in measuring sarcomere lengths and tendon slack lengths in vivo in muscles throughout the lower limb, improving these data was not possible here, but does not lessen the impact of the results presented. Nevertheless, obtaining in vivo estimates of sarcomere lengths through micro‐endoscope imaging (Chen and Delp, 2016; Chen *et al*., 2016) or tendon slack lengths through elastography (Hug *et al*., 2013) for implementations into musculoskeletal models is a possible area of future study and opportunity to improve this subject‐specific modelling framework.

Regarding the construction of the musculoskeletal models built here, the musculotendon unit attachment points were placed based on volumetric muscle meshes from digital segmentation of T1 MR images, which maximises the potential to accurately represent average muscle paths. However, the single‐line actuators often used to represent single muscles within multi‐body dynamics models may not be sufficient in reflecting in vivo anatomy at certain areas of the lower limb. It is possible that this is particularly important in the pelvis/hip region, where muscle paths and wrapping can be particularly complex (Lenaerts *et al*., 2008; Modenese *et al*., 2011; Modenese and Kohout, 2020), and failure to capture this complexity in the models created here could explain the high average RMSE values seen during hip extension/flexion movements in all models. Future implementations of the methods presented here could involve imaging the muscles of interest multiple times with the lower limb in different postures, which could allow for the visualisation of how muscles or even individual fibres wrap around and interact with other musculoskeletal structures. Increasing joint complexity or the anatomical accuracy of the MTU actuator models used to represent each muscle (Modenese *et al*., 2011; Modenese and Kohout, 2020) may reduce these errors; however, doing so was out of the scope of this study.

While the data presented here suggest that the subject‐specific models were on average the most accurate relative to their generic equivalents across all joint tested, their predictions of muscle torques were not perfect and, in some cases, resulted in similar RMSE values than the generic young models. Therefore, this should not entirely discourage the use of scaled generic or even generic muscle properties in subject‐specific models in all contexts, particularly if obtaining detailed individualised data is not possible. Indeed, generic‐based models of the vertebrate musculoskeletal system have formed the foundations of the biomechanical modelling field for many years and have shown to be of great use in many clinical and non‐clinical studies (Delp *et al*., 1990; Delp and Zajac, 1992; Delp *et al*., 1995; Herrmann and Delp, 1999; Murray *et al*., 2002; Erdemir and Piazza, 2004; Hutchinson *et al*., 2005; Steele *et al*., 2012; O'Neill *et al*., 2013; Hicks *et al*., 2015; Hutchinson *et al*., 2015; Seth *et al*., 2018; Falisse *et al*., 2019; Imani Nejad *et al*., 2020). Ultimately, given the increased time and monetary costs associated with creating subject‐specific musculoskeletal models relative to generic modelling, the goal and impact of a given study should dictate the degree of individualisation which is needed within the musculoskeletal models. However, for novel studies into form–function relationships within the musculoskeletal system, the high accuracy and the inherent advantages provided by subject‐specific models and simulations mean that they could become a gold standard and form the basis for future studies into how muscle functions relate to subject‐specific anatomy.

## CONFLICT OF INTEREST

The authors have no competing interests to declare.

## AUTHOR CONTRIBUTIONS

JPC, KTB and KD conceived the study; JPC and BG collected the isokinetic dynamometer data; BG recruited the participants; JPC processed MR images, built the models, ran the simulations and analysed the resulting data; all authors critically revised the manuscript and approved the final version for publication; KTB and KD secured funding for the project.

## ETHICAL APPROVAL

All participants for this study were recruited in accordance with ethical approval from the University of Liverpool's Central University Research Ethics Committee for Physical Interventions (Reference number: 3757).

## Supporting information

App S1

## Data Availability

All raw data (including magnetic resonance images and isokinetic dynamometer data), data at key stages of processing and final data (including subject‐specific models) produced here will be available online at University of Liverpool's Research Data Catalogue (DataCat; http://datacat.liverpool.ac.uk/1105/). [Correction added on 2 July 2020, after first online publication: The data availability link has been added.]
