# Supplementary material for: Subject‐specific muscle properties from diffusion tensor imaging significantly improve the accuracy of musculoskeletal models
Source: J Anat. 2020 Jun 29;237(5):941–59. doi: 10.1111/joa.13261 (PMC7542200; doi:10.1111/joa.13261)
Supplement: Supplementary file 1 — App S1 [file JOA-237-941-s001.docx]

**Appendix S1**

**Reserve actuators**

For each model (Subject-specific- SS, Generic Elderly- GE, Generic Elderly Optimised -GE_O_, Generic Young- GY, Generic Young Optimised- GY_O_), each static optimisation simulation was repeated twice, with and without any reserve actuators appended to the unlocked degree of freedom (ankle plantarflexion-dorsiflexion, knee extension-flexion, hip extension-flexion). These actuators can be applied to musculoskeletal models and simulations in order to compensate for unmodelled structures or potential errors in model construction (muscle force generating properties or musculoskeletal geometry), but are highly penalised by the cost function of the optimisation algorithm. Therefore, they are only recruited by the optimisation if the musculotendon unit models are not strong enough to satisfy the applied external forces. So the relative forces generating by these actuators in each model type can be compared to assess their abilities to accurately simulate muscle functional capabilities. For simulations with reserve actuators included, the optimal force was set to 5% of the max net joint moment as predicted by inverse dynamics (calculated using the reserve actuation tool with Opensim [1], which is in line with recommended values for reserve actuator forces in musculoskeletal models and simulations [1].

When reserve actuators were applied to the simulations, the GY, GY_O_ and SS models required the least degree of reserve actuation during most of the simulated movements (S1 Fig). The GE models required peak reserve actuator forces of 121% and 96.1% of max joint moment during ankle plantarflexion and dorsiflexion respectively, while the GE_O_ models on average needed peak of 26.6% and 34.9% (S1 Fig). Similar reserve actuator torques were required at the ankle in the GY models (25.4% and 31.8%), but these reduced to 21.4% and 15.9% in the GY_O_ models. The SS models required relatively minimal reserve actuation throughout, with a peak of 7.8% of max joint moment during the entire rotation (Fig S1 A). Similar trends were seen throughout knee extension and flexion, with the SS models requiring peak actuator forces of 15.7% and 14.6% of max joint moment respectively, compared to 85.5% and 21.3% in the GE models and 29.8% and 21.7% in the GE_O_ models. Reserve actuator force during knee extension was smaller in the GY models compared to the SS models (12.2%), but larger in the GY_O_ models (25%). The reserve actuator forces in the GY and GY_O_ models during knee flexion were similar to the SS models (15% and 15.7% respectively) (Fig. S1 B). More reserve actuation was needed during hip extension and flexion in all the models, with peaks of 14.7% and 24.8% of max moment in the SS models. Similar actuation was needed in the GY (29% and 24%) and GY_O_ models (14% and 20%). These however were considerably smaller than the GE and GE_O_ models, which required forces of 50.8% and 41.3% respectively during hip extension, and 54.4% and 54.5% during hip flexion (Fig S1 C).

The low degree of reserve actuation required by the SS, GY and GY_O_ models, particularly relative to the GE and GE_O_ models, suggest that they simulate the desired maximal effort movements, and therefore the associated muscle functional outputs, to the greatest degree of “anatomical accuracy” (Fig S1). It is generally accepted within OpenSim musculoskeletal modelling literature that reserve actuator forces should be around or below 10% of maximum joint moment for the simulation to be considered valid [1]. Reserve actuator forces in the SS, GY and GY_O_ models were below or around this value throughout all simulated movements, with the exception of hip flexion. This suggests that musculoskeletal models of young, healthy individuals which contain muscle properties from the same individual, or individuals of a similar age demographic, are more anatomically and dynamically “realistic” than those with elderly, cadaveric muscle architecture, as suggested by the differences in muscle force generating properties shown in Figure 2.

**
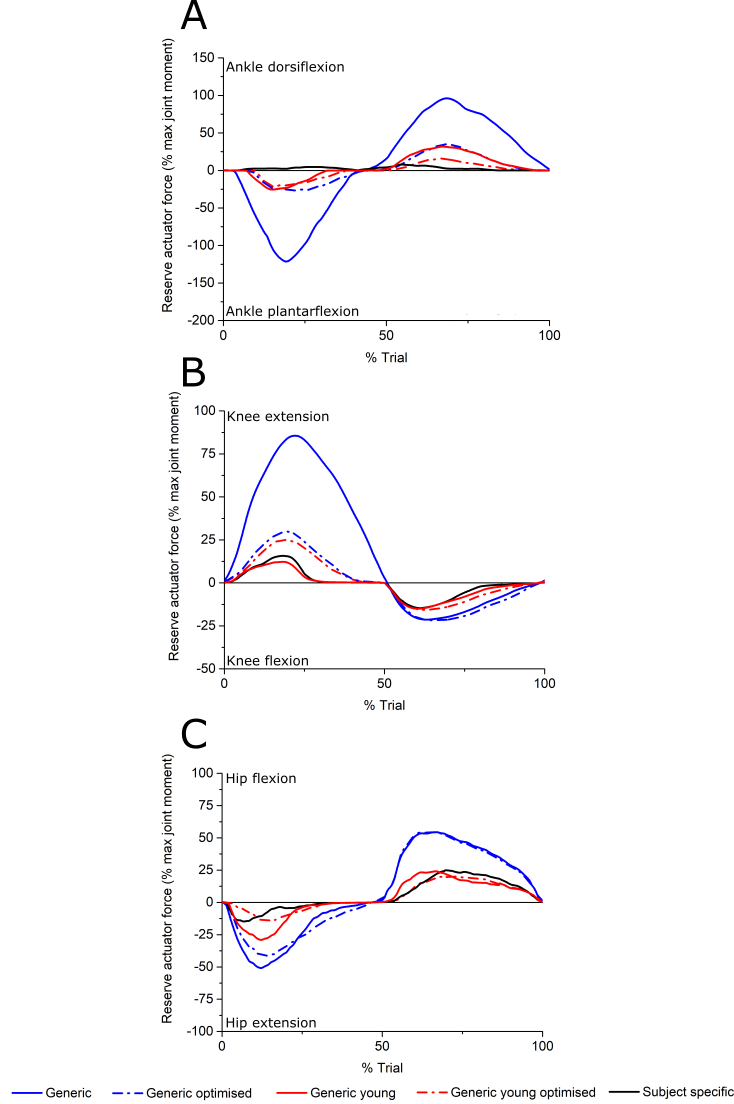
**

**Figure S1.** Reserve actuator forces (expressed as % of maximum joint moment) through static optimisation simulations of ankle plantarflexion-dorsiflexion (A), knee extension-flexion (B) and hip extension-flexion (C).

**Table S1. Study participant information**

| Subject no. | Sex | Age | Height (cm) | Body mass (kg) | BMI (kgm^-2^) |
| --- | --- | --- | --- | --- | --- |
| 1 | M | 35 | 176 | 68 | 21.95 |
| 2 | M | 32 | 182 | 74.7 | 22.55 |
| 3 | F | 26 | 176 | 72.6 | 23.44 |
| 4 | M | 23 | 180 | 77.4 | 23.89 |
| 5 | F | 33 | 165 | 60.6 | 22.26 |
| 6 | M | 29 | 177 | 68.9 | 21.99 |
| 7 | F | 32 | 168 | 53.7 | 19.03 |
| 8 | M | 28 | 186 | 83.3 | 24.08 |
| 9 | F | 26 | 164 | 53.5 | 20.01 |
| 10 | F | 29 | 180 | 66 | 20.37 |

**Table S2. Muscles included in each model their respective functional group classifications.** Functional groupings were based on previous muscle architecture studies [2, 3] and also informed by muscle moment arms from the musculoskeletal models created here. * All knee flexors except Sartorius were also categorised as hip extensors for the purpose of the static optimisation simulations.

| **Functional group** | **Muscle** | **Abbreviation** |
| --- | --- | --- |
| Hip extensors | Gluteus maximus | GMax |
|  | Gluteus medius | GMed |
|  | Gluteus minimus | GMin |
|  | Piriformis | PIRI |
| Hip flexors | Iliacus | ILI |
|  | Psaos | PMA |
| Hip adductors | Adductor magnus | AM |
|  | Adductor longus | AL |
|  | Adductor brevis | AB |
|  | Pectineus | PECT |
| Knee flexors/ Hip extensors | Gracilis | GRA |
|  | Semimembranosus | SM |
|  | Semitendinosus | ST |
|  | Biceps femoris- long head | BFL |
|  | Biceps femoris- short head | BFS |
|  | Sartorius* | SAR |
| Knee extensors | Rectus femoris | RF |
|  | Vastus lateralis | VL |
|  | Vastus medialis | VM |
|  | Vastus intermedius | VI |
| Ankle dorsiflexors | Tibialis anterior | TA |
|  | Extensor digitorum longus | EDL |
|  | Extensor hallucis longus | EHL |
| Ankle plantarflexors | Medial gastrocnemius | MG |
|  | Lateral gastrocnemius | LG |
|  | Soleus | SOL |
|  | Tibialis posterior | TP |
|  | Flexor digitorum longus | FDL |
|  | Peroneus longus | PL |
|  | Peroneus brevis | PB |
|  | Flexor hallucis longus | FHL |

**Table S3. Wrapping object properties within each subject-specific musculoskeletal model.**

| **Wrap object name** | **Shape** | **Location** | **Muscles wrapped** |
| --- | --- | --- | --- |
| Hip flexors at pelvis | Cylinder | Anterior aspect of pubic ramus | Iliacus  Psoas |
| Knee extensors at femur | Cylinder | Anterior aspect of femoral condyles | Rectus femoris  Vastus lateralis  Vastus medialis  Vastus intermedius |
| Gastrocs at tibia | Cylinder | Posterior aspect of proximal tibia | Medial gastrocnemius  Lateral gastrocnemius |
| Hamstring at tibia | Sphere | Medial aspect of proximal tibia | Semimembranosus  Semitendinosus  Gracilis  Sartorius |

Table S4. Muscle force generating properties within the subject-specific lower limb musculoskeletal model of Subject 1, obtained from magnetic resonance imaging and diffusion tensor imaging. For muscle abbreviation definitions, see Table 1. L_f_- muscle fibre length. L_ts_- tendon slack length.

| **MTU** | **Max force** | **L_f_** | **Pennation** | **L_ts_** |
| --- | --- | --- | --- | --- |
| **GMax 1** | 954 | 0.085 | 0.38 | 0.163 |
| **GMax 2** | 954 | 0.085 | 0.38 | 0.183 |
| **GMax 3** | 954 | 0.085 | 0.38 | 0.184 |
| **GMed 1** | 276 | 0.103 | 0.36 | 0.066 |
| **GMed 2** | 276 | 0.103 | 0.36 | 0.066 |
| **GMed 3** | 276 | 0.103 | 0.36 | 0.026 |
| **GMin 1** | 125 | 0.046 | 0.36 | 0.030 |
| **GMin 2** | 125 | 0.046 | 0.36 | 0.023 |
| **GMin 3** | 125 | 0.046 | 0.36 | 0.022 |
| **PIRI** | 171 | 0.047 | 0.36 | 0.040 |
| **ILI** | 887 | 0.051 | 0.25 | 0.218 |
| **PMA** | 1822 | 0.032 | 0.19 | 0.340 |
| **AM 1** | 443 | 0.220 | 0.27 | 0.184 |
| **AM 2** | 443 | 0.220 | 0.27 | 0.075 |
| **AL** | 952 | 0.050 | 0.12 | 0.178 |
| **AB** | 745 | 0.028 | 0.11 | 0.109 |
| **PECT** | 202 | 0.030 | 0.51 | 0.080 |
| **GRA** | 146 | 0.190 | 0.14 | 0.222 |
| **SM** | 790 | 0.072 | 0.23 | 0.365 |
| **ST** | 647 | 0.103 | 0.26 | 0.340 |
| **BFL** | 535 | 0.103 | 0.20 | 0.330 |
| **BFS** | 513 | 0.044 | 0.21 | 0.251 |
| **SAR** | 97 | 0.405 | 0.00 | 0.167 |
| **RF** | 679 | 0.095 | 0.24 | 0.408 |
| **VL** | 1397 | 0.103 | 0.32 | 0.365 |
| **VM** | 1157 | 0.097 | 0.52 | 0.335 |
| **VI** | 1561 | 0.105 | 0.08 | 0.320 |
| **TA** | 887 | 0.054 | 0.17 | 0.314 |
| **EDL** | 1025 | 0.026 | 0.19 | 0.470 |
| **EHL** | 478 | 0.014 | 0.16 | 0.361 |
| **MG** | 1502 | 0.041 | 0.17 | 0.364 |
| **LG** | 671 | 0.062 | 0.21 | 0.325 |
| **SOL** | 1243 | 0.103 | 0.49 | 0.271 |
| **TP** | 879 | 0.031 | 0.24 | 0.355 |
| **FDL** | 413 | 0.021 | 0.24 | 0.446 |
| **PL** | 989 | 0.035 | 0.25 | 0.405 |
| **PB** | 317 | 0.027 | 0.20 | 0.235 |
| **FHL** | 1158 | 0.039 | 0.29 | 0.390 |

| **MTU** | **Max force** | **L_f_** | **Pennation** | **L_ts_** |
| --- | --- | --- | --- | --- |
| **GMax 1** | 836 | 0.105 | 0.60 | 0.143 |
| **GMax 2** | 836 | 0.105 | 0.60 | 0.173 |
| **GMax 3** | 836 | 0.105 | 0.60 | 0.233 |
| **GMed 1** | 376 | 0.101 | 0.41 | 0.176 |
| **GMed 2** | 376 | 0.101 | 0.41 | 0.176 |
| **GMed 3** | 376 | 0.101 | 0.41 | 0.176 |
| **GMin 1** | 380 | 0.043 | 0.36 | 0.069 |
| **GMin 2** | 380 | 0.043 | 0.36 | 0.069 |
| **GMin 3** | 380 | 0.043 | 0.36 | 0.069 |
| **PIRI** | 249 | 0.061 | 0.36 | 0.120 |
| **ILI** | 1677 | 0.027 | 0.86 | 0.237 |
| **PMA** | 780 | 0.051 | 0.79 | 0.269 |
| **AM 1** | 870 | 0.103 | 0.46 | 0.234 |
| **AM 2** | 870 | 0.103 | 0.46 | 0.305 |
| **AL** | 1409 | 0.044 | 0.66 | 0.238 |
| **AB** | 602 | 0.055 | 0.61 | 0.139 |
| **PECT** | 210 | 0.029 | 0.50 | 0.083 |
| **GRA** | 115 | 0.260 | 0.14 | 0.152 |
| **SM** | 1571 | 0.078 | 0.22 | 0.340 |
| **ST** | 630 | 0.076 | 0.28 | 0.300 |
| **BFL** | 573 | 0.130 | 0.26 | 0.280 |
| **BFS** | 330 | 0.092 | 0.26 | 0.155 |
| **SAR** | 99 | 0.375 | 0.00 | 0.140 |
| **RF** | 637 | 0.114 | 0.32 | 0.453 |
| **VL** | 2173 | 0.094 | 0.47 | 0.420 |
| **VM** | 1213 | 0.105 | 0.48 | 0.370 |
| **VI** | 1861 | 0.089 | 0.40 | 0.408 |
| **TA** | 879 | 0.049 | 0.36 | 0.300 |
| **EDL** | 715 | 0.041 | 0.35 | 0.460 |
| **EHL** | 188 | 0.018 | 0.24 | 0.290 |
| **MG** | 1284 | 0.057 | 0.49 | 0.410 |
| **LG** | 591 | 0.080 | 0.23 | 0.350 |
| **SOL** | 1089 | 0.146 | 0.38 | 0.270 |
| **TP** | 576 | 0.053 | 0.39 | 0.310 |
| **FDL** | 289 | 0.017 | 0.45 | 0.460 |
| **PL** | 751 | 0.052 | 0.40 | 0.400 |
| **PB** | 240 | 0.029 | 0.27 | 0.300 |
| **FHL** | 535 | 0.041 | 0.50 | 0.400 |

Table S5. Muscle force generating properties within the subject-specific lower limb musculoskeletal model of Subject 2, obtained from magnetic resonance imaging and diffusion tensor imaging. For muscle abbreviation definitions, see Table 1. L_f_- muscle fibre length. L_ts_- tendon slack length.

Table S6. Muscle force generating properties within the subject-specific lower limb musculoskeletal model of Subject 3, obtained from magnetic resonance imaging and diffusion tensor imaging. For muscle abbreviation definitions, see Table 1. L_f_- muscle fibre length. L_ts_- tendon slack length.

| **MTU** | **Max force** | **L_f_** | **Pennation** | **L_ts_** |
| --- | --- | --- | --- | --- |
| **GMax 1** | 600 | 0.078 | 0.59 | 0.150 |
| **GMax 2** | 600 | 0.078 | 0.59 | 0.190 |
| **GMax 3** | 600 | 0.078 | 0.59 | 0.190 |
| **GMed 1** | 184 | 0.101 | 0.61 | 0.070 |
| **GMed 2** | 184 | 0.101 | 0.61 | 0.070 |
| **GMed 3** | 184 | 0.101 | 0.61 | 0.070 |
| **GMin 1** | 146 | 0.043 | 0.65 | 0.060 |
| **GMin 2** | 146 | 0.043 | 0.65 | 0.060 |
| **GMin 3** | 146 | 0.043 | 0.65 | 0.060 |
| **PIRI** | 168 | 0.047 | 0.36 | 0.080 |
| **ILI** | 1332 | 0.024 | 0.67 | 0.230 |
| **PMA** | 490 | 0.034 | 0.63 | 0.269 |
| **AM 1** | 388 | 0.145 | 0.34 | 0.243 |
| **AM 2** | 388 | 0.145 | 0.34 | 0.283 |
| **AL** | 775 | 0.031 | 0.59 | 0.070 |
| **AB** | 646 | 0.040 | 0.47 | 0.150 |
| **PECT** | 190 | 0.025 | 0.53 | 0.075 |
| **GRA** | 141 | 0.186 | 0.14 | 0.202 |
| **SM** | 451 | 0.105 | 0.33 | 0.264 |
| **ST** | 382 | 0.096 | 0.27 | 0.265 |
| **BFL** | 311 | 0.110 | 0.24 | 0.280 |
| **BFS** | 328 | 0.052 | 0.38 | 0.180 |
| **SAR** | 128 | 0.411 | 0.00 | 0.127 |
| **RF** | 798 | 0.066 | 0.42 | 0.470 |
| **VL** | 1707 | 0.074 | 0.47 | 0.410 |
| **VM** | 1496 | 0.057 | 0.41 | 0.360 |
| **VI** | 1199 | 0.071 | 0.38 | 0.410 |
| **TA** | 540 | 0.049 | 0.24 | 0.339 |
| **EDL** | 434 | 0.036 | 0.27 | 0.480 |
| **EHL** | 197 | 0.026 | 0.33 | 0.400 |
| **MG** | 1498 | 0.025 | 0.67 | 0.370 |
| **LG** | 756 | 0.040 | 0.36 | 0.370 |
| **SOL** | 978 | 0.104 | 0.43 | 0.260 |
| **TP** | 350 | 0.052 | 0.43 | 0.330 |
| **FDL** | 193 | 0.024 | 0.28 | 0.460 |
| **PL** | 414 | 0.052 | 0.34 | 0.400 |
| **PB** | 91 | 0.027 | 0.35 | 0.290 |
| **FHL** | 406 | 0.026 | 0.40 | 0.380 |

Table S7. Muscle force generating properties within the subject-specific lower limb musculoskeletal model of Subject 4, obtained from magnetic resonance imaging and diffusion tensor imaging. For muscle abbreviation definitions, see Table 1. L_f_- muscle fibre length. L_ts_- tendon slack length.

| **MTU** | **Max force** | **L_f_** | **Pennation** | **L_ts_** |
| --- | --- | --- | --- | --- |
| **GMax 1** | 610 | 0.150 | 0.71 | 0.100 |
| **GMax 2** | 610 | 0.150 | 0.71 | 0.100 |
| **GMax 3** | 610 | 0.150 | 0.71 | 0.130 |
| **GMed 1** | 393 | 0.078 | 0.59 | 0.100 |
| **GMed 2** | 393 | 0.078 | 0.59 | 0.100 |
| **GMed 3** | 393 | 0.078 | 0.59 | 0.100 |
| **GMin 1** | 252 | 0.046 | 0.68 | 0.060 |
| **GMin 2** | 252 | 0.046 | 0.68 | 0.060 |
| **GMin 3** | 252 | 0.046 | 0.68 | 0.060 |
| **PIRI** | 452 | 0.030 | 0.39 | 0.100 |
| **ILI** | 810 | 0.062 | 0.43 | 0.190 |
| **PMA** | 1380 | 0.056 | 0.45 | 0.330 |
| **AM 1** | 800 | 0.136 | 0.48 | 0.200 |
| **AM 2** | 800 | 0.136 | 0.48 | 0.280 |
| **AL** | 914 | 0.075 | 0.41 | 0.190 |
| **AB** | 588 | 0.049 | 0.48 | 0.140 |
| **PECT** | 213 | 0.039 | 0.59 | 0.070 |
| **GRA** | 176 | 0.167 | 0.14 | 0.270 |
| **SM** | 988 | 0.080 | 0.45 | 0.380 |
| **ST** | 684 | 0.088 | 0.23 | 0.380 |
| **BFL** | 399 | 0.174 | 0.30 | 0.270 |
| **BFS** | 362 | 0.067 | 0.45 | 0.370 |
| **SAR** | 123 | 0.385 | 0.00 | 0.130 |
| **RF** | 933 | 0.081 | 0.32 | 0.440 |
| **VL** | 2713 | 0.107 | 0.42 | 0.360 |
| **VM** | 1331 | 0.123 | 0.49 | 0.300 |
| **VI** | 1104 | 0.116 | 0.32 | 0.330 |
| **TA** | 570 | 0.072 | 0.37 | 0.300 |
| **EDL** | 532 | 0.051 | 0.30 | 0.450 |
| **EHL** | 148 | 0.025 | 0.56 | 0.330 |
| **MG** | 706 | 0.082 | 0.41 | 0.400 |
| **LG** | 660 | 0.067 | 0.37 | 0.360 |
| **SOL** | 1112 | 0.128 | 0.36 | 0.230 |
| **TP** | 518 | 0.068 | 0.28 | 0.310 |
| **FDL** | 279 | 0.030 | 0.50 | 0.420 |
| **PL** | 411 | 0.070 | 0.34 | 0.370 |
| **PB** | 181 | 0.040 | 0.21 | 0.260 |
| **FHL** | 376 | 0.069 | 0.27 | 0.320 |

Table S8. Muscle force generating properties within the subject-specific lower limb musculoskeletal model of Subject 5, obtained from magnetic resonance imaging and diffusion tensor imaging. For muscle abbreviation definitions, see Table 1. L_f_- muscle fibre length. L_ts_- tendon slack length.

| **MTU** | **Max force** | **L_f_** | **Pennation** | **L_ts_** |
| --- | --- | --- | --- | --- |
| **GMax 1** | 481 | 0.131 | 0.64 | 0.080 |
| **GMax 2** | 481 | 0.131 | 0.64 | 0.130 |
| **GMax 3** | 481 | 0.131 | 0.64 | 0.180 |
| **GMed 1** | 342 | 0.068 | 0.63 | 0.100 |
| **GMed 2** | 342 | 0.068 | 0.63 | 0.090 |
| **GMed 3** | 342 | 0.068 | 0.63 | 0.070 |
| **GMin 1** | 246 | 0.028 | 0.83 | 0.090 |
| **GMin 2** | 246 | 0.028 | 1.83 | 0.080 |
| **GMin 3** | 246 | 0.028 | 2.83 | 0.080 |
| **PIRI** | 212 | 0.039 | 0.66 | 0.100 |
| **ILI** | 912 | 0.037 | 0.47 | 0.200 |
| **PMA** | 951 | 0.038 | 0.46 | 0.300 |
| **AM 1** | 601 | 0.115 | 0.63 | 0.210 |
| **AM 2** | 601 | 0.115 | 0.63 | 0.290 |
| **AL** | 647 | 0.071 | 0.35 | 0.140 |
| **AB** | 499 | 0.059 | 0.47 | 0.090 |
| **PECT** | 245 | 0.041 | 0.50 | 0.060 |
| **GRA** | 131 | 0.171 | 0.14 | 0.250 |
| **SM** | 597 | 0.093 | 0.40 | 0.350 |
| **ST** | 734 | 0.063 | 0.24 | 0.400 |
| **BFL** | 498 | 0.129 | 0.36 | 0.320 |
| **BFS** | 547 | 0.061 | 0.42 | 0.380 |
| **SAR** | 89 | 0.370 | 0.00 | 0.140 |
| **RF** | 715 | 0.086 | 0.22 | 0.380 |
| **VL** | 2183 | 0.103 | 0.40 | 0.360 |
| **VM** | 1070 | 0.106 | 0.56 | 0.330 |
| **VI** | 943 | 0.119 | 0.37 | 0.330 |
| **TA** | 548 | 0.075 | 0.28 | 0.275 |
| **EDL** | 572 | 0.040 | 0.28 | 0.400 |
| **EHL** | 121 | 0.043 | 0.37 | 0.260 |
| **MG** | 848 | 0.069 | 0.44 | 0.320 |
| **LG** | 870 | 0.045 | 0.27 | 0.330 |
| **SOL** | 786 | 0.152 | 0.38 | 0.200 |
| **TP** | 636 | 0.051 | 0.35 | 0.290 |
| **FDL** | 177 | 0.043 | 0.22 | 0.375 |
| **PL** | 659 | 0.041 | 0.29 | 0.390 |
| **PB** | 105 | 0.050 | 0.19 | 0.260 |
| **FHL** | 208 | 0.124 | 0.46 | 0.320 |

Table S9. Muscle force generating properties within the subject-specific lower limb musculoskeletal model of Subject 6, obtained from magnetic resonance imaging and diffusion tensor imaging. For muscle abbreviation definitions, see Table 1. L_f_- muscle fibre length. L_ts_- tendon slack length.

| **MTU** | **Max force** | **L_f_** | **Pennation** | **L_ts_** |
| --- | --- | --- | --- | --- |
| **GMax 1** | 499 | 0.118 | 0.74 | 0.170 |
| **GMax 2** | 499 | 0.118 | 0.74 | 0.220 |
| **GMax 3** | 499 | 0.118 | 0.74 | 0.230 |
| **GMed 1** | 411 | 0.077 | 0.54 | 0.100 |
| **GMed 2** | 411 | 0.077 | 0.54 | 0.100 |
| **GMed 3** | 411 | 0.077 | 0.54 | 0.080 |
| **GMin 1** | 172 | 0.048 | 0.93 | 0.070 |
| **GMin 2** | 172 | 0.048 | 0.93 | 0.070 |
| **GMin 3** | 172 | 0.048 | 0.93 | 0.070 |
| **PIRI** | 210 | 0.042 | 0.41 | 0.080 |
| **ILI** | 592 | 0.057 | 0.46 | 0.140 |
| **PMA** | 599 | 0.065 | 0.41 | 0.235 |
| **AM 1** | 527 | 0.131 | 0.35 | 0.250 |
| **AM 2** | 527 | 0.131 | 0.35 | 0.280 |
| **AL** | 1002 | 0.048 | 0.62 | 0.180 |
| **AB** | 609 | 0.041 | 0.64 | 0.100 |
| **PECT** | 163 | 0.059 | 0.47 | 0.030 |
| **GRA** | 154 | 0.182 | 0.14 | 0.220 |
| **SM** | 514 | 0.093 | 0.38 | 0.360 |
| **ST** | 272 | 0.138 | 0.28 | 0.300 |
| **BFL** | 317 | 0.158 | 0.31 | 0.280 |
| **BFS** | 490 | 0.069 | 0.35 | 0.380 |
| **SAR** | 119 | 0.395 | 0.00 | 0.140 |
| **RF** | 566 | 0.120 | 0.39 | 0.400 |
| **VL** | 1464 | 0.150 | 0.45 | 0.340 |
| **VM** | 969 | 0.138 | 0.47 | 0.330 |
| **VI** | 923 | 0.099 | 0.31 | 0.390 |
| **TA** | 558 | 0.103 | 0.26 | 0.300 |
| **EDL** | 618 | 0.046 | 0.28 | 0.485 |
| **EHL** | 278 | 0.041 | 0.27 | 0.410 |
| **MG** | 1152 | 0.068 | 0.34 | 0.400 |
| **LG** | 579 | 0.070 | 0.33 | 0.390 |
| **SOL** | 842 | 0.148 | 0.36 | 0.230 |
| **TP** | 674 | 0.061 | 0.37 | 0.330 |
| **FDL** | 346 | 0.039 | 0.39 | 0.465 |
| **PL** | 489 | 0.067 | 0.19 | 0.400 |
| **PB** | 91 | 0.041 | 0.18 | 0.235 |
| **FHL** | 719 | 0.056 | 0.33 | 0.410 |

Table S10. Muscle force generating properties within the subject-specific lower limb musculoskeletal model of Subject 7, obtained from magnetic resonance imaging and diffusion tensor imaging. For muscle abbreviation definitions, see Table 1. L_f_- muscle fibre length. L_ts_- tendon slack length.

| **MTU** | **Max force** | **L_f_** | **Pennation** | **L_ts_** |
| --- | --- | --- | --- | --- |
| **GMax 1** | 654 | 0.061 | 0.72 | 0.230 |
| **GMax 2** | 654 | 0.061 | 0.72 | 0.230 |
| **GMax 3** | 654 | 0.061 | 0.72 | 0.230 |
| **GMed 1** | 377 | 0.036 | 0.94 | 0.093 |
| **GMed 2** | 377 | 0.036 | 0.94 | 0.093 |
| **GMed 3** | 377 | 0.036 | 0.94 | 0.093 |
| **GMin 1** | 141 | 0.053 | 0.63 | 0.069 |
| **GMin 2** | 141 | 0.053 | 0.63 | 0.069 |
| **GMin 3** | 141 | 0.053 | 0.63 | 0.069 |
| **PIRI** | 166 | 0.051 | 0.36 | 0.120 |
| **ILI** | 592 | 0.043 | 0.50 | 0.160 |
| **PMA** | 1015 | 0.041 | 0.56 | 0.238 |
| **AM 1** | 804 | 0.059 | 0.58 | 0.280 |
| **AM 2** | 804 | 0.059 | 0.58 | 0.320 |
| **AL** | 887 | 0.034 | 0.62 | 0.238 |
| **AB** | 926 | 0.025 | 0.72 | 0.170 |
| **PECT** | 210 | 0.044 | 0.54 | 0.050 |
| **GRA** | 121 | 0.190 | 0.14 | 0.180 |
| **SM** | 626 | 0.107 | 0.47 | 0.290 |
| **ST** | 297 | 0.107 | 0.23 | 0.320 |
| **BFL** | 365 | 0.107 | 0.32 | 0.300 |
| **BFS** | 350 | 0.043 | 0.24 | 0.150 |
| **SAR** | 100 | 0.345 | 0.00 | 0.147 |
| **RF** | 533 | 0.094 | 0.37 | 0.423 |
| **VL** | 1221 | 0.084 | 0.42 | 0.380 |
| **VM** | 574 | 0.092 | 0.66 | 0.360 |
| **VI** | 1563 | 0.056 | 0.39 | 0.380 |
| **TA** | 601 | 0.042 | 0.40 | 0.330 |
| **EDL** | 530 | 0.035 | 0.38 | 0.550 |
| **EHL** | 236 | 0.013 | 0.32 | 0.320 |
| **MG** | 800 | 0.053 | 0.39 | 0.330 |
| **LG** | 660 | 0.033 | 0.33 | 0.330 |
| **SOL** | 1024 | 0.087 | 0.46 | 0.260 |
| **TP** | 584 | 0.037 | 0.46 | 0.290 |
| **FDL** | 219 | 0.022 | 0.40 | 0.490 |
| **PL** | 523 | 0.042 | 0.38 | 0.360 |
| **PB** | 133 | 0.028 | 0.36 | 0.230 |
| **FHL** | 278 | 0.059 | 0.39 | 0.330 |

| **MTU** | **Max force** | **L_f_** | **Pennation** | **L_ts_** |
| --- | --- | --- | --- | --- |
| **GMax 1** | 634 | 0.142 | 0.64 | 0.100 |
| **GMax 2** | 634 | 0.142 | 0.64 | 0.150 |
| **GMax 3** | 634 | 0.142 | 0.64 | 0.170 |
| **GMed 1** | 403 | 0.142 | 0.64 | 0.100 |
| **GMed 2** | 403 | 0.142 | 0.64 | 0.090 |
| **GMed 3** | 403 | 0.142 | 0.64 | 0.070 |
| **GMin 1** | 210 | 0.142 | 0.64 | 0.060 |
| **GMin 2** | 210 | 0.142 | 0.64 | 0.070 |
| **GMin 3** | 210 | 0.142 | 0.64 | 0.070 |
| **PIRI** | 489 | 0.027 | 0.83 | 0.140 |
| **ILI** | 862 | 0.053 | 0.44 | 0.180 |
| **PMA** | 1555 | 0.056 | 0.49 | 0.300 |
| **AM 1** | 723 | 0.137 | 0.54 | 0.200 |
| **AM 2** | 723 | 0.137 | 0.54 | 0.300 |
| **AL** | 1075 | 0.068 | 0.25 | 0.140 |
| **AB** | 562 | 0.053 | 0.54 | 0.090 |
| **PECT** | 481 | 0.044 | 0.30 | 0.040 |
| **GRA** | 151 | 0.186 | 0.14 | 0.260 |
| **SM** | 508 | 0.094 | 0.38 | 0.400 |
| **ST** | 568 | 0.067 | 0.20 | 0.400 |
| **BFL** | 319 | 0.191 | 0.37 | 0.250 |
| **BFS** | 302 | 0.115 | 0.26 | 0.330 |
| **SAR** | 107 | 0.437 | 0.00 | 0.150 |
| **RF** | 1122 | 0.076 | 0.26 | 0.450 |
| **VL** | 2551 | 0.093 | 0.40 | 0.400 |
| **VM** | 1146 | 0.134 | 0.45 | 0.310 |
| **VI** | 1014 | 0.131 | 0.42 | 0.340 |
| **TA** | 486 | 0.103 | 0.30 | 0.320 |
| **EDL** | 972 | 0.035 | 0.26 | 0.520 |
| **EHL** | 210 | 0.041 | 0.27 | 0.425 |
| **MG** | 1058 | 0.077 | 0.47 | 0.460 |
| **LG** | 666 | 0.072 | 0.40 | 0.390 |
| **SOL** | 958 | 0.182 | 0.41 | 0.400 |
| **TP** | 663 | 0.051 | 0.27 | 0.330 |
| **FDL** | 253 | 0.034 | 0.38 | 0.450 |
| **PL** | 871 | 0.049 | 0.41 | 0.460 |
| **PB** | 194 | 0.056 | 0.28 | 0.285 |
| **FHL** | 347 | 0.085 | 0.30 | 0.390 |

Table S11. Muscle force generating properties within the subject-specific lower limb musculoskeletal model of Subject 8, obtained from magnetic resonance imaging and diffusion tensor imaging. For muscle abbreviation definitions, see Table 1. L_f_- muscle fibre length. L_ts_- tendon slack length.

Table S12. Muscle force generating properties within the subject-specific lower limb musculoskeletal model of Subject 9, obtained from magnetic resonance imaging and diffusion tensor imaging. For muscle abbreviation definitions, see Table 1. L_f_- muscle fibre length. L_ts_- tendon slack length.

| **MTU** | **Max force** | **L_f_** | **Pennation** | **L_ts_** |
| --- | --- | --- | --- | --- |
| **GMax 1** | 353 | 0.125 | 0.60 | 0.110 |
| **GMax 2** | 353 | 0.125 | 0.60 | 0.190 |
| **GMax 3** | 353 | 0.125 | 0.60 | 0.200 |
| **GMed 1** | 285 | 0.059 | 0.67 | 0.080 |
| **GMed 2** | 285 | 0.059 | 0.67 | 0.080 |
| **GMed 3** | 285 | 0.059 | 0.67 | 0.060 |
| **GMin 1** | 116 | 0.041 | 0.82 | 0.070 |
| **GMin 2** | 116 | 0.041 | 0.82 | 0.060 |
| **GMin 3** | 116 | 0.041 | 0.82 | 0.050 |
| **PIRI** | 150 | 0.041 | 0.19 | 0.100 |
| **ILI** | 563 | 0.046 | 0.42 | 0.120 |
| **PMA** | 261 | 0.102 | 0.30 | 0.230 |
| **AM 1** | 302 | 0.145 | 0.33 | 0.110 |
| **AM 2** | 302 | 0.145 | 0.33 | 0.210 |
| **AL** | 417 | 0.055 | 0.85 | 0.140 |
| **AB** | 362 | 0.061 | 0.41 | 0.080 |
| **PECT** | 323 | 0.030 | 0.54 | 0.070 |
| **GRA** | 111 | 0.186 | 0.14 | 0.220 |
| **SM** | 609 | 0.082 | 0.28 | 0.310 |
| **ST** | 460 | 0.080 | 0.16 | 0.330 |
| **BFL** | 340 | 0.139 | 0.30 | 0.250 |
| **BFS** | 356 | 0.048 | 0.16 | 0.330 |
| **SAR** | 70 | 0.437 | 0.00 | 0.080 |
| **RF** | 636 | 0.071 | 0.28 | 0.370 |
| **VL** | 1575 | 0.091 | 0.38 | 0.310 |
| **VM** | 535 | 0.134 | 0.40 | 0.240 |
| **VI** | 673 | 0.118 | 0.36 | 0.280 |
| **TA** | 439 | 0.067 | 0.28 | 0.280 |
| **EDL** | 442 | 0.052 | 0.22 | 0.400 |
| **EHL** | 111 | 0.032 | 0.24 | 0.320 |
| **MG** | 743 | 0.066 | 0.34 | 0.320 |
| **LG** | 433 | 0.063 | 0.22 | 0.320 |
| **SOL** | 596 | 0.158 | 0.36 | 0.180 |
| **TP** | 302 | 0.064 | 0.38 | 0.265 |
| **FDL** | 171 | 0.022 | 0.31 | 0.335 |
| **PL** | 594 | 0.054 | 0.37 | 0.350 |
| **PB** | 110 | 0.039 | 0.31 | 0.220 |
| **FHL** | 447 | 0.036 | 0.27 | 0.435 |

Table S13. Muscle force generating properties within the subject-specific lower limb musculoskeletal model of Subject 10, obtained from magnetic resonance imaging and diffusion tensor imaging. For muscle abbreviation definitions, see Table 1. L_f_- muscle fibre length. L_ts_- tendon slack length.

| **MTU** | **Max force** | **L_f_** | **Pennation** | **L_ts_** |
| --- | --- | --- | --- | --- |
| **GMax 1** | 427 | 0.131 | 0.73 | 0.110 |
| **GMax 2** | 427 | 0.131 | 0.73 | 0.165 |
| **GMax 3** | 427 | 0.131 | 0.73 | 0.180 |
| **GMed 1** | 401 | 0.061 | 0.54 | 0.100 |
| **GMed 2** | 401 | 0.061 | 0.54 | 0.110 |
| **GMed 3** | 401 | 0.061 | 0.54 | 0.100 |
| **GMin 1** | 148 | 0.020 | 0.82 | 0.080 |
| **GMin 2** | 148 | 0.020 | 0.82 | 0.085 |
| **GMin 3** | 148 | 0.020 | 0.82 | 0.085 |
| **PIRI** | 215 | 0.039 | 0.41 | 0.100 |
| **ILI** | 731 | 0.046 | 0.42 | 0.192 |
| **PMA** | 742 | 0.049 | 0.32 | 0.332 |
| **AM 1** | 298 | 0.155 | 0.40 | 0.150 |
| **AM 2** | 298 | 0.155 | 0.40 | 0.260 |
| **AL** | 638 | 0.055 | 0.32 | 0.175 |
| **AB** | 295 | 0.071 | 0.51 | 0.090 |
| **PECT** | 125 | 0.025 | 0.41 | 0.040 |
| **GRA** | 92 | 0.175 | 0.14 | 0.275 |
| **SM** | 619 | 0.080 | 0.33 | 0.350 |
| **ST** | 381 | 0.093 | 0.19 | 0.340 |
| **BFL** | 316 | 0.148 | 0.33 | 0.280 |
| **BFS** | 300 | 0.059 | 0.31 | 0.375 |
| **SAR** | 95 | 0.394 | 0.00 | 0.170 |
| **RF** | 623 | 0.082 | 0.23 | 0.425 |
| **VL** | 1573 | 0.089 | 0.36 | 0.380 |
| **VM** | 746 | 0.092 | 0.21 | 0.305 |
| **VI** | 651 | 0.113 | 0.28 | 0.340 |
| **TA** | 584 | 0.047 | 0.26 | 0.315 |
| **EDL** | 762 | 0.022 | 0.29 | 0.445 |
| **EHL** | 146 | 0.032 | 0.29 | 0.330 |
| **MG** | 484 | 0.077 | 0.47 | 0.350 |
| **LG** | 463 | 0.054 | 0.43 | 0.370 |
| **SOL** | 517 | 0.138 | 0.36 | 0.230 |
| **TP** | 349 | 0.064 | 0.36 | 0.300 |
| **FDL** | 218 | 0.016 | 0.55 | 0.427 |
| **PL** | 523 | 0.052 | 0.31 | 0.375 |
| **PB** | 99 | 0.042 | 0.28 | 0.240 |
| **FHL** | 198 | 0.078 | 0.26 | 0.392 |

**Table S14. Isokinetic dynamometer settings for measuring isokinetic and isometric muscle torques around the ankle, knee and hip joints.** For the isokinetic trials, joint angles represent the mean ranges of motion from full dorsiflexion/flexion to full plantarflexion/extension across all subjects.

| **Joint** | **Movement** | **Rotational speed (°s^-1^)** | **Joint angle** | **Repetitions (rest)** |
| --- | --- | --- | --- | --- |
| Ankle | Isokinetic- Plantarflexion/ dorsiflexion | 30 | -16° - 42° | 5 |
|  | Isometric- Plantarflexion | N/A | 0° | 5 (5 seconds) |
|  | Isometric- Dorsiflexion | N/A | 25° plantarflexion | 5 (5 seconds) |
| Knee | Isokinetic- Extension/flexion | 60 | 111° - -3° | 5 |
|  | Isometric- Extension | N/A | 60° flexion | 5 (5 seconds) |
|  | Isometric- Flexion | N/A | 0° | 5 (5 seconds) |
| Hip | Isokinetic- Extension/flexion | 30 | 111° - 0° | 5 |
|  | Isometric- Extension | N/A | 45° flexion | 5 (5 seconds) |
|  | Isometric- Flexion | N/A | 45° flexion | 5 (5 seconds) |

Table S15. Root Mean Squared errors, expressed as % of maximum torque measured from the isokinetic dynamometer (Te), of predicted muscle torques from simulations of isokinetic trials at the ankle, knee and hip joints in Subject-specific (SS), Generic young (GY), Generic young optimised (GYo), Generic (GE) and Generic optimised (GEO) musculoskeletal models of subjects 1-5. Results from trials with no reserve actuators and with reserve actuator force (F_act_) set to 5% of predicted net joint moment are shown.

|  | | Root mean squared error (% Max T_e_) | | | | | | | | | | | |
| --- | --- | --- | --- | --- | --- | --- | --- | --- | --- | --- | --- | --- | --- |
| Joint | | Ankle | | | | Knee | | | | Hip | | | |
|  | Motion | Plantarflexion | | Dorsiflexion | | Extension | | Flexion | | Extension | | Flexion | |
| Subject | _Model_ ^Fact^ | 0 | 5 | 0 | 5 | 0 | 5 | 0 | 5 | 0 | 5 | 0 | 5 |
| S01 | SS | 10.3 | 10.3 | 2.5 | 2.9 | 7.3 | 6.8 | 0.0 | 2.9 | 27.5 | 28.0 | 41.1 | 41.2 |
|  | GY | 9.9 | 9.9 | 34.9 | 35.0 | 6.7 | 6.2 | 2.9 | 7.3 | 21.4 | 21.6 | 4.7 | 5.1 |
|  | GY_O_ | 11.6 | 11.7 | 28.2 | 28.4 | 24.9 | 24.8 | 31.5 | 33.3 | 33.5 | 33.6 | 9.5 | 10.0 |
|  | GE | 42.9 | 42.9 | 62.7 | 62.8 | 64.8 | 64.8 | 22.5 | 25.8 | 37.9 | 38.0 | 54.3 | 54.4 |
|  | GE_O_ | 11.3 | 11.4 | 43.6 | 43.8 | 41.5 | 41.5 | 44.5 | 46.3 | 56.0 | 56.1 | 44.4 | 44.4 |
| S02 | SS | 16.4 | 16.4 | 11.7 | 11.8 | 11.6 | 11.3 | 10.3 | 13.9 | 13.9 | 15.2 | 28.7 | 28.8 |
|  | GY | 17.1 | 17.1 | 23.9 | 24.0 | 17.5 | 17.4 | 25.0 | 28.1 | 33.0 | 33.5 | 25.9 | 26.1 |
|  | GY_O_ | 16.7 | 16.7 | 12.4 | 12.6 | 38.0 | 38.0 | 55.2 | 56.4 | 28.5 | 29.2 | 48.5 | 48.5 |
|  | GE | 18.2 | 18.3 | 62.4 | 62.4 | 62.1 | 62.2 | 43.5 | 45.6 | 40.6 | 41.4 | 46.2 | 46.4 |
|  | GE_O_ | 16.9 | 17.0 | 22.4 | 22.6 | 47.0 | 47.0 | 63.9 | 65.1 | 42.7 | 43.3 | 54.3 | 54.4 |
| S03 | SS | 22.1 | 22.1 | 15.2 | 15.3 | 18.8 | 18.7 | 20.6 | 21.8 | 21.4 | 22.0 | 3.9 | 4.3 |
|  | GY | 22.4 | 22.4 | 4.7 | 4.8 | 19.9 | 19.9 | 12.3 | 12.8 | 21.1 | 21.3 | 3.5 | 3.3 |
|  | GY_O_ | 21.9 | 21.9 | 1.3 | 1.4 | 19.8 | 19.6 | 15.1 | 15.5 | 24.3 | 24.4 | 25.9 | 26.1 |
|  | GE | 23.8 | 23.8 | 54.4 | 54.6 | 32.4 | 32.5 | 18.5 | 19.1 | 28.8 | 29.0 | 39.0 | 39.2 |
|  | GE_O_ | 22.2 | 22.2 | 4.6 | 4.8 | 19.2 | 18.9 | 16.5 | 17.3 | 24.7 | 25.1 | 38.1 | 38.3 |
| S04 | SS | 8.7 | 8.7 | 3.4 | 3.5 | 17.7 | 17.6 | 21.6 | 23.4 | 16.2 | 16.5 | 43.8 | 43.8 |
|  | GY | 8.8 | 8.9 | 6.3 | 6.5 | 13.3 | 13.3 | 36.5 | 37.7 | 25.2 | 25.7 | 46.3 | 46.3 |
|  | GY_O_ | 8.9 | 9.0 | 12.0 | 12.2 | 8.0 | 7.9 | 30.0 | 31.6 | 18.0 | 18.5 | 41.2 | 41.4 |
|  | GE | 9.1 | 9.2 | 51.7 | 52.0 | 65.6 | 65.7 | 50.4 | 51.4 | 33.3 | 33.6 | 61.6 | 61.6 |
|  | GE_O_ | 9.2 | 9.4 | 28.5 | 28.6 | 27.1 | 27.1 | 46.7 | 47.9 | 23.0 | 23.4 | 54.8 | 54.8 |
| S05 | SS | 5.8 | 5.8 | 0.2 | 0.1 | 8.5 | 8.5 | 13.3 | 16.2 | 8.6 | 9.4 | 24.1 | 25.0 |
|  | GY | 5.8 | 5.8 | 0.9 | 1.1 | 7.6 | 7.6 | 15.5 | 18.1 | 8.3 | 8.5 | 4.5 | 5.4 |
|  | GY_O_ | 7.6 | 7.6 | 9.5 | 9.8 | 10.9 | 10.9 | 25.1 | 27.7 | 23.9 | 24.1 | 43.9 | 44.5 |
|  | GE | 35.5 | 35.5 | 32.9 | 33.1 | 60.0 | 60.1 | 42.5 | 44.6 | 24.6 | 24.7 | 35.3 | 36.0 |
|  | GE_O_ | 18.2 | 18.2 | 22.2 | 22.4 | 26.3 | 26.3 | 40.5 | 42.9 | 38.2 | 38.2 | 49.9 | 50.1 |

|  | | Root mean squared error (% Max T_e_) | | | | | | | | | | | |
| --- | --- | --- | --- | --- | --- | --- | --- | --- | --- | --- | --- | --- | --- |
| Joint | | Ankle | | | | Knee | | | | Hip | | | |
|  | Motion | Plantarflexion | | Dorsiflexion | | Extension | | Flexion | | Extension | | Flexion | |
| Subject | _Model_ ^Fact^ | 0 | 5 | 0 | 5 | 0 | 5 | 0 | 5 | 0 | 5 | 0 | 5 |
| S06 | SS | 10.5 | 10.5 | 1.8 | 2.0 | 6.8 | 6.6 | 18.5 | 20.0 | 21.9 | 22.6 | 44.6 | 44.1 |
|  | GY | 29.1 | 29.1 | 32.2 | 32.2 | 5.2 | 4.9 | 27.5 | 28.7 | 23.5 | 23.9 | 42.4 | 42.3 |
|  | GY_O_ | 28.5 | 28.5 | 12.0 | 12.1 | 4.8 | 4.3 | 20.5 | 22.0 | 17.7 | 18.5 | 16.9 | 17.4 |
|  | GE | 48.4 | 48.4 | 62.2 | 62.4 | 61.0 | 61.0 | 42.3 | 43.4 | 35.4 | 35.7 | 51.5 | 51.5 |
|  | GE_O_ | 35.9 | 35.9 | 30.2 | 30.3 | 18.6 | 18.5 | 34.9 | 36.4 | 29.6 | 29.9 | 49.2 | 49.3 |
| S07 | SS | 8.7 | 8.7 | 3.1 | 3.0 | 4.2 | 4.5 | 26.2 | 27.5 | 26.9 | 26.0 | 13.8 | 13.0 |
|  | GY | 8.8 | 8.8 | 4.6 | 4.8 | 4.5 | 4.6 | 7.0 | 8.7 | 30.6 | 30.7 | 36.2 | 36.2 |
|  | GY_O_ | 13.2 | 13.2 | 3.3 | 3.4 | 4.7 | 4.7 | 20.6 | 22.0 | 19.9 | 20.1 | 67.1 | 67.2 |
|  | GE | 38.0 | 38.0 | 63.4 | 63.5 | 57.1 | 57.2 | 23.4 | 25.2 | 40.9 | 41.0 | 48.2 | 48.4 |
|  | GE_O_ | 9.0 | 9.0 | 12.8 | 12.9 | 5.1 | 5.2 | 42.1 | 42.9 | 40.4 | 40.5 | 58.1 | 58.1 |
| S08 | SS | 10.0 | 10.0 | 17.0 | 17.1 | 30.0 | 30.0 | 7.8 | 11.7 | 34.6 | 34.8 | 16.6 | 15.3 |
|  | GY | 10.4 | 10.6 | 54.4 | 54.4 | 24.4 | 24.4 | 31.9 | 34.8 | 35.2 | 35.6 | 6.2 | 5.9 |
|  | GY_O_ | 11.1 | 11.2 | 22.3 | 22.5 | 7.5 | 7.3 | 7.6 | 11.6 | 34.0 | 34.1 | 19.7 | 18.4 |
|  | GE | 33.0 | 33.0 | 61.3 | 61.4 | 59.6 | 59.6 | 47.0 | 49.3 | 42.4 | 42.9 | 55.6 | 55.8 |
|  | GE_O_ | 12.2 | 12.4 | 37.2 | 37.4 | 21.0 | 20.9 | 18.4 | 22.8 | 34.6 | 34.9 | 37.0 | 37.1 |
| S09 | SS | 14.2 | 14.2 | 17.9 | 17.9 | 12.4 | 12.4 | 9.7 | 10.3 | 21.5 | 21.6 | 30.7 | 30.8 |
|  | GY | 13.3 | 13.3 | 16.1 | 16.1 | 10.7 | 10.5 | 4.8 | 5.1 | 15.5 | 15.7 | 11.3 | 10.9 |
|  | GY_O_ | 14.7 | 14.7 | 6.2 | 6.0 | 57.8 | 57.8 | 16.0 | 16.3 | 17.0 | 17.4 | 2.3 | 2.5 |
|  | GE | 14.9 | 14.9 | 2.6 | 2.4 | 59.1 | 59.2 | 16.2 | 16.8 | 16.6 | 17.5 | 24.6 | 24.5 |
|  | GE_O_ | 19.2 | 19.3 | 0.3 | 0.4 | 9.9 | 9.6 | 10.0 | 11.5 | 20.0 | 20.8 | 42.6 | 42.9 |
| S10 | SS | 9.4 | 9.4 | 1.4 | 1.6 | 14.7 | 14.6 | 15.0 | 21.3 | 27.0 | 27.2 | 2.8 | 3.4 |
|  | GY | 9.2 | 9.2 | 10.6 | 10.5 | 5.5 | 5.3 | 12.5 | 15.9 | 25.3 | 25.5 | 22.0 | 21.7 |
|  | GY_O_ | 10.6 | 10.7 | 1.6 | 1.4 | 5.6 | 5.4 | 13.0 | 15.7 | 27.9 | 28.0 | 18.3 | 17.9 |
|  | GE | 9.4 | 9.4 | 40.2 | 40.4 | 51.9 | 52.0 | 19.2 | 24.8 | 27.8 | 28.4 | 41.5 | 41.7 |
|  | GE_O_ | 12.0 | 12.0 | 14.0 | 14.2 | 5.2 | 4.8 | 15.5 | 23.3 | 27.3 | 27.5 | 48.9 | 49.0 |

Table S16. Root Mean Squared errors, expressed as % of maximum torque measured from the isokinetic dynamometer (Te), of predicted muscle torques from simulations of isokinetic trials at the ankle, knee and hip joints in Subject-specific (SS), Generic young (GY), Generic young optimised (GY_O_), Generic elderly (GE) and generic elderly optimised (GE_O_) musculoskeletal models of subjects 6-10. Results from trials with no reserve actuators and with reserve actuator force (F_act_) set to 5% of predicted net joint moment are shown.

Table S17. Statistical significance levels of comparisons between root mean squared errors of mean predicted isokinetic muscle torques at the ankle, knee and hip joints relative to experimentally measured muscle torques in the Generic elderly (GE), Generic elderly optimised (GE_O_), Generic young (GY), Generic young optimised (GY_O_) and Subject-specific (SS) models. Italicised numbers in bold indicate statistically significant differences (p<0.05).

| **Joint** | **Movement** | **Model** | **GE** | **GEO** | **GY** | **GYO** | **SS** |
| --- | --- | --- | --- | --- | --- | --- | --- |
| **Ankle** | **Plantarflexion** | **GE** | N/A | 0.06 | ***0.007*** | ***0.016*** | ***0.002*** |
|  |  | **GE_O_** | 0.06 | N/A | 0.92 | 0.98 | 0.70 |
|  |  | **GY** | ***0.007*** | 0.92 | N/A | 0.99 | 0.98 |
|  |  | **GY_O_** | ***0.016*** | 0.98 | 0.99 | N/A | 0.94 |
|  |  | **SS** | ***0.002*** | 0.70 | 0.98 | 0.94 | N/A |
|  | **Dorsiflexion** | **GE** | N/A | ***<0.001*** | ***<0.001*** | ***<0.001*** | ***<0.001*** |
|  |  | **GE_O_** | ***<0.001*** | N/A | 0.99 | 0.44 | 0.18 |
|  |  | **GY** | ***<0.001*** | 0.99 | N/A | 0.71 | 0.37 |
|  |  | **GY_O_** | ***<0.001*** | 0.44 | 0.71 | N/A | 0.98 |
|  |  | **SS** | ***<0.001*** | 0.18 | 0.37 | 0.98 | N/A |
| **Knee** | **Extension** | **GE** | N/A | ***<0.001*** | ***<0.001*** | ***<0.001*** | ***<0.001*** |
|  |  | **GE_O_** | ***<0.001*** | N/A | 0.29 | 0.94 | 0.45 |
|  |  | **GY** | ***<0.001*** | 0.29 | N/A | 0.72 | 0.99 |
|  |  | **GY_O_** | ***<0.001*** | 0.94 | 0.72 | N/A | 0.88 |
|  |  | **SS** | ***<0.001*** | 0.45 | 0.99 | 0.88 | N/A |
|  | **Flexion** | **GE** | N/A | 0.99 | 0.10 | 0.53 | ***0.02*** |
|  |  | **GE_O_** | 0.99 | N/A | 0.07 | 0.46 | ***0.01*** |
|  |  | **GY** | 0.10 | 0.07 | N/A | 0.85 | 0.98 |
|  |  | **GY_O_** | 0.53 | 0.46 | 0.85 | N/A | 0.53 |
|  |  | **SS** | ***0.02*** | ***0.01*** | 0.98 | 0.53 | N/A |
| **Hip** | **Extension** | **GE** | N/A | 0.99 | 0.14 | 0.18 | ***0.04*** |
|  |  | **GE_O_** | 0.99 | N/A | 0.08 | 0.12 | ***0.02*** |
|  |  | **GY** | 0.14 | 0.08 | N/A | 0.99 | 0.98 |
|  |  | **GY_O_** | 0.18 | 0.12 | 0.99 | N/A | 0.99 |
|  |  | **SS** | ***0.04*** | ***0.02*** | 0.98 | 0.99 | N/A |
|  | **Flexion** | **GE** | N/A | 0.99 | ***0.003*** | 0.11 | ***0.02*** |
|  |  | **GE_O_** | 0.99 | N/A | ***0.001*** | 0.06 | ***0.01*** |
|  |  | **GY** | ***0.002*** | ***0.001*** | N/A | 0.65 | 0.95 |
|  |  | **GY_O_** | ***0.001*** | ***0.04*** | 0.79 | N/A | 0.96 |
|  |  | **SS** | ***0.02*** | ***0.01*** | 0.95 | 0.99 | N/A |

[1] Hicks, J.L., Uchida, T.K., Seth, A., Rajagopal, A. & Delp, S.L. 2015 Is my model good enough? Best practices for verification and validation of musculoskeletal models and simulations of movement. *J Biomech Eng* **137**, 020905. (doi:10.1115/1.4029304).

[2] Ward, S.R., Eng, C.M., Smallwood, L.H. & Lieber, R.L. 2009 Are current measurements of lower extremity muscle architecture accurate? *Clin Orthop Relat Res* **467**, 1074-1082. (doi:10.1007/s11999-008-0594-8).

[3] Charles, J.P., Suntaxi, F. & Anderst, W.J. 2019 In vivo human lower limb muscle architecture dataset obtained using diffusion tensor imaging. *PLoS One* **14**, e0223531. (doi:10.1371/journal.pone.0223531).
